# Use of cyclic peptides to induce crystallization: case study with prolyl hydroxylase domain 2

**DOI:** 10.1038/s41598-020-76307-8

**Published:** 2020-12-15

**Authors:** Rasheduzzaman Chowdhury, Martine I. Abboud, Tom E. McAllister, Biswadip Banerji, Bhaskar Bhushan, John L. Sorensen, Akane Kawamura, Christopher J. Schofield

**Affiliations:** 1 Chemistry Research Laboratory, Department of Chemistry, University of Oxford, Oxford, OX1 3TA UK; 2 Chemistry – School of Natural and Environmental Sciences, Newcastle University, Newcastle upon Tyne, NE1 7RU UK

**Keywords:** NMR spectroscopy, X-ray crystallography, Oxidoreductases, Peptides, Chemical biology

## Abstract

Crystallization is the bottleneck in macromolecular crystallography; even when a protein crystallises, crystal packing often influences ligand-binding and protein–protein interaction interfaces, which are the key points of interest for functional and drug discovery studies. The human hypoxia-inducible factor prolyl hydroxylase 2 (PHD2) readily crystallises as a homotrimer, but with a sterically blocked active site. We explored strategies aimed at altering PHD2 crystal packing by protein modification and molecules that bind at its active site and elsewhere. Following the observation that, despite weak inhibition/binding in solution, succinamic acid derivatives readily enable PHD2 crystallization, we explored methods to induce crystallization *without* active site binding. Cyclic peptides obtained via mRNA display bind PHD2 tightly away from the active site. They efficiently enable PHD2 crystallization in different forms, both with/without substrates, apparently by promoting oligomerization involving binding to the *C*-terminal region. Although our work involves a specific case study, together with those of others, the results suggest that mRNA display-derived cyclic peptides may be useful in challenging protein crystallization cases.

## Introduction

X-ray diffraction analysis of proteins and their complexes is a mainstay of modern biological sciences and medicinal chemistry, yet protein crystallization, in particular of forms reflecting the solution state, is often a stumbling block in biophysical studies. Multiple strategies have been explored to stabilise proteins/protein complexes and/or to reduce the protein conformational heterogeneity, which in general hinders crystallization. Such strategies include, but are not limited to, protein construct design^1^, reduction of surface entropy^2^, co-complexation with natural/therapeutic ligands/chemical probes^3^ and binding of auxiliary proteins such as antibody fragments or alternative scaffolds^4–7^. Here we describe our experience in crystallizing a challenging human protein target and how this led us to a non-standard strategy to obtain different crystal forms. To obtain robust crystallization conditions for hypoxia-inducible factor prolyl hydroxylase 2 (PHD2) in complex with inhibitors and substrates, we explored all of the aforementioned approaches, but none has been efficient to date. As an alternative, we explored the use of cyclic peptides (CPs), which we identified via application of mRNA display technology^8–10^; this led to a CP that binds tightly to the catalytic domain of PHD2 (*K*_D_ value of 270 pM)^8^ at a site away from the active site in a manner that efficiently enables efficient crystallization in previously unobserved forms.

In animals, the α,β-heterodimeric hypoxia-inducible factors (HIFs) activate an array of genes including those encoding for vascular endothelial growth factor (VEGF) and erythropoietin (EPO), which work to ameliorate the effects of hypoxia^11,12^. Under normoxic conditions, HIFα subunits are rapidly destroyed via ubiquitination involving an E3 ligase complex (von Hippel Lindau protein, Elongins B and C, Cul2 and Rbx1) and proteasomes^13,14^. The degradation of HIFα subunits is promoted by post-translational hydroxylation of prolyl-residues located in its *N*/*C*-terminal oxygen dependent degradation domains (NODD and CODD)^15,16^. These dioxygen-sensitive reactions are catalysed by 2-oxoglutarate (2OG) and Fe(II) dependent prolyl hydroxylases (PHD 1–3 in humans)^11,12,17^. Pharmacological manipulation of the hypoxic response via manipulating PHD activity offers the possibility of treating tumours and ischemia related diseases. Small-molecule PHD inhibitors are approved/in clinical trials for the treatment of anaemia associated with chronic kidney diseases^18,19^, which is presently treated with erythropoietin (EPO), a common medicine for anaemia^20^.

The development of clinically useful PHD inhibitors has been enabled by structural studies. Crystal structures of the PHD2 catalytic domain (aa 181–426) were initially reported in complex with a transition metal ion and bicyclic inhibitors (which are related to FG2216, a PHD inhibitor that entered clinical trials) in the *P*6_3_ crystal form^21,22^. Subsequently PHD2-ligand complex structures, including with 2OG (PDB: 3OUJ), have been reported in different space groups^23,24^. The structural work to date has employed PHD2 constructs truncated at the *C*-terminus, i.e. aa 181–392 which crystallises in the *P*2_1_2_1_2_1_ space group (PDB: 3OUI)^24^, aa 189–399 in *P*6_3_ (PDB: 4JZR)^25^, aa 181–416 in both *P*6_3_ (PDB: 3OUH)^24^ and *P*4_1_ (PDB: 3OUJ)^24^, aa 184–419 (PDB: 4KBZ) and aa 181–416 (PDB: 5V18)^23^ in *P*4_1_ forms. Collectively, these observations imply that interactions involving the *C*-terminal region of PHD2 and binding of specific ligands to the active site can have profound roles in enabling crystallization/different crystal packing of PHD2.

**Figure 1:**
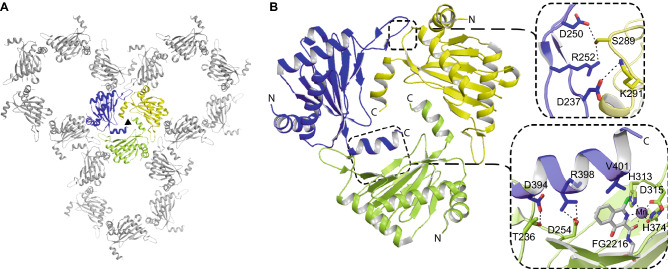
PHD2 crystallizes as a homotrimer with bicyclic ligands. (**A**) The homotrimeric crystal packing observed in the nPHD2/*P*6_3_ crystal structure (PDB ID: 4BQX). (**B**) The PHD2 homotrimer is stabilised by intermolecular interactions between the active site residues including from the *N*-terminal β2/β3 loop (aa 237–254) and the *C*-terminal helix α4 (aa 393–402) of a threefold symmetry related molecule. The overall PHD2 (aa 188–404) fold consists of the major (β1, β8, β5, β10, β4) and minor (β7, β6, β9, β-II) β-sheets of the DSBH, and four α helices. Both the β2/β3 ‘finger’ loop and the *C*-terminal helix α4 of the PHDs directly interact with the HIF-α ODDs (see Fig. 4).

Although PHD2 is predominantly monomeric in solution, crystallographic analyses in the *P*6_3_ form reveal homotrimeric organization with intermolecular interactions between the residues from *C*-terminal helix α4 of one monomer and the surrounding active site residues from the flexible β2/β3 loop of a neighbouring subunit^21,22^ (Fig. 1). This head-to-tail trimeric arrangement^21,22^ prevents the substrate from productively approaching the active site in the *P*6_3_ crystal form. Disrupting the head-to-tail arrangement by engineering *C*-terminal residues that are directly involved in crystal packing enables crystallization of PHD2-ODD enzyme–substrate complexes (space group: *P*2_1_2_1_2_1_)^26,27^. However, this procedure has two limitations: compared to the *P*6_3_ form, only very few crystals were obtained via removing such ‘unwanted’ oligomerization, thereby limiting investigations, such as analysing catalytic intermediates using time-resolved crystallography. Further, this crystallization process takes an extended period (months) during which time crystallization drops can become contaminated (e.g. by Gram-negative *Stenotrophomonas *spp., sometimes leading to orthorhombic crystals of *Stenotrophomonas* alkaline phosphatase^28^).

With the aim of developing new PHD2 crystal forms that may enable a ligand accessible active site, i.e. one free from crystal packing/conformational restraints, that is amenable to ligand and/or substrate binding, we explored diverse strategies to promote crystallization, including variation of active site ligands, manipulating PHD2 surface solvation/interactions by lysine-*N*^ε^-methylation^29^, and a non-standard method using non active site binding CPs. Here we report a methodology for the efficient crystallization of PHD2 complexes, with and without HIF-α ODD substrate present, by using tight binding reagents, i.e. CPs, which allow retention of catalytic activity, but which dramatically promote crystallization of PHD2 complexes.

## Results

### PHD2 crystallization can be induced by some ligands that bind weakly in solution

Following from the observation that specific heteroaromatic inhibitors with glycine side chains induce PHD2 crystallization in the *P*6_3_ form^21,22^, we investigated the selectivity of small-molecule induced PHD2 crystallization. We employed 96-well screens for crystallizing PHD2 constructs (PHD2_181-426_ and PHD2_181-407_, hereafter nPHD2 and cPHD2, respectively) with a set of cyclic 2OG analogues and related small-molecules. We observed that crystallization of nPHD2 is induced by succinamic acid derivatives (SCAs), **4a**, **4b, 7f.** and **30a** (Fig. S1). This is interesting because none of these SCAs are potent PHD2 inhibitors or stabilizers (as observed by MS, NMR and thermal shift analyses) in solution (Figs. S1, S2). Except for the nPHD2.**4b** complex, which crystallized in the *P*3_1_ form, all the aforementioned SCAs crystallized with nPHD2 in the *P*6_3_ form. The nPHD2.SCA complex structures manifest a similar ligand binding mode to that observed in the reported nPHD2.FG2216/*P*6_3_ structures^21,22^, i.e. like FG2216 and related compounds, the SCAs coordinate the active site metal ion via their heteroaromatic ring nitrogen and side chain amide carbonyl oxygen (Fig. S1). However, while the bidentate coordination of the active site metal ion by SCAs forms an approximately planar six-membered chelate ring, more established PHD inhibitors, including FG2216, form a five-membered chelate ring (Fig. S1). The chelate ring size may affect the stability of the protein-complex^30^ and possibly its binding/inhibition potential. Although biophysical (MS, NMR) analyses suggest that SCAs form only a weak complex with monomeric nPHD2 (Fig. S2), the clear *F*_o_-*F*_c_ difference density for the SCAs suggests that intermolecular interactions of SCAs notably with the *C*-terminus of a threefold symmetry related neighbour in the *P*6_3_ form may both stabilize the ligand binding in the crystal lattice and help promote crystallization.

### Surface methylation of PHD2

The results with small molecules prompted us to investigate other ways of inducing PHD2 crystallization. We modified the lysine residues (21) in the nPHD2 construct via reductive lysine-*N*^ε^-methylation, which has been used to change crystal packing contacts for crystallization of other proteins^2,29^ but not iron oxygenases such as the PHDs. Electrospray ionization mass spectrometry (ESI–MS) under denaturing conditions of the methylation product (nPHD2-Me) revealed masses corresponding to multiple dimethylation events, apparently of all 21 lysines plus at the *N*-terminal amine. We confirmed the methylation states by mass spectrometric (MS) analysis following trypsinolysis of nPHD2-Me; the so obtained LC–MS spectra differ from those of unmodified PHD2 implying ‘missed cleavages’. LC–MS/MS analyses on the fragments of nPHD2-Me provided MS/MS evidence for *N*-dimethylation of lysines (data not shown).

nPHD2-Me (rather unexpectedly) crystallized in a hexagonal rod morphology in complex with an SCA, **4a**. Despite modifications of all 21 lysines in nPHD2, the structure was solved in the same form, i.e. the *P*6_3_ form, as that of unmodified PHD2 with **4a**/related ligands. Comparison of the nPHD2 and nPHD2-Me structures reveals very similar overall folds, with the β2/β3 ‘finger’ loop (that is involved in HIF ODD binding^26,27^) in an ‘open’ (i.e. non-productive) conformation in both cases (Fig. S3)^21,27^. Although lysine methylation did not alter the packing of nPHD2 crystals, some of the lysines including K216, K262, K291, K350, K400 and K402 became more ordered upon methylation as apparent in their difference (*F*_o_-*F*_c_) density maps (Fig. S3).

Because some of the *N*^ε^-methylated lysines in nPHD2-Me are involved in HIF-α ODD binding^26,27^, we tested the activity of nPHD2-Me by monitoring HIF-1α ODD hydroxylation and 2OG turnover (Fig. S3). Despite some evidence for modulation of 2OG turnover, the results show that under the tested conditions, nPHD2-Me has substantial catalytic activity with both CODD or NODD (Fig. S3). Overall, these results imply that the differences in surface chemistry induced by *N*^ε^-methylation of lysines are insufficient to enable the desired changes in crystallization.

### Crystallization of PHD2 with cyclic peptides (CPs)

Together with previous observations^22,24,25^, the results presented here support the proposal that nPHD2 often preferentially crystallizes in the *P*6_3_ form, independent of different types of chemical modifications or crystallization conditions. We therefore set out to investigate the crystallization potential of PHD2 surface-binders that form contacts in the crystalline lattice, but which do not cause loss of catalytic activity.

With this objective in mind, we investigated peptides binding to nPHD2, which were identified using the mRNA display based Random nonstandard Peptide Integrated Discovery (RaPID) method that has been used to identify both inhibitory and allosteric CPs, including ones selective for enzymes involved in signalling pathways and transcriptional regulation^8,31,32^. In the RaPID method, acyclic peptides are cyclized by S_N_2 reaction of a *C*-terminal cysteine and a *N*-terminal chloroacetyl group to give a thioether^9^. As described previously, we identified peptides binding tightly to PHD2 as revealed by NMR and other biophysical analyses, but which do not affect Fe(II)/2OG/substrate binding and which allow efficient catalysis (we term these ‘non-competitive CPs’, NCCPs)^8^. Previously reported crystal structures of RaPID derived CPs in complex with KDM4A (a 2OG-oxygenase involved in epigenetic regulation)^31^ and Semaphorin 4D Receptor Plexin B1 (a signalling protein)^33^ reveal that in both cases, the target-selective CPs bind at the active site and adopt a distorted β-sheet fold with β-turns at the ends of the CP. Because the edges of β-sheets are intrinsically prone to undergo β H-bonding with other β-strands, as manifested in fibrils, and β-rich proteins^34,35^, we reasoned that the NCCPs, which can be readily prepared by routine solid phase peptide synthesis, might enable ordered oligomerization leading to crystallization of PHD2.

**Figure 2:**
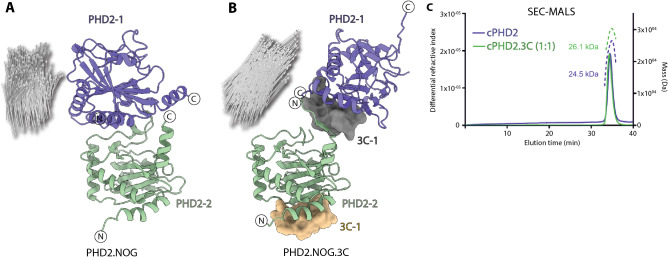
Comparison of the PHD2.NOG and PHD2.NOG.3C complex structures reveals binding of 3C away from the active site. The figure shows views from crystal structures of PHD2.NOG complexes alone (**A**) or in complex with 3C (**B**). The cPHD2.NOG.3C structure was solved in the *P*6_5_ space group and has a single molecule in the asymmetric unit. 3C slots in between two symmetrically related PHD2 monomers in the crystal lattice, making intermolecular backbone-to-backbone interactions involving β1 (aa 204–214) and three residues towards the *N*-terminus (187–189) within the same protein monomer (PHD2-2) and six residues (aa 399–404) at the *C*-terminus of another monomer (PHD2-1). Grey arrows indicate directional vectors from PHD2-1 residues to PHD2-2 in normal (**A**) and 3C-induced (**B**) crystal packing. (**c**) SEC-MALS analysis of cPHD2 with and without 3C reveals that 3C-induced oligomerization applies only in crystals as cPHD2 is predominantly monomeric even in the presence of 3C.

**Figure 3:**
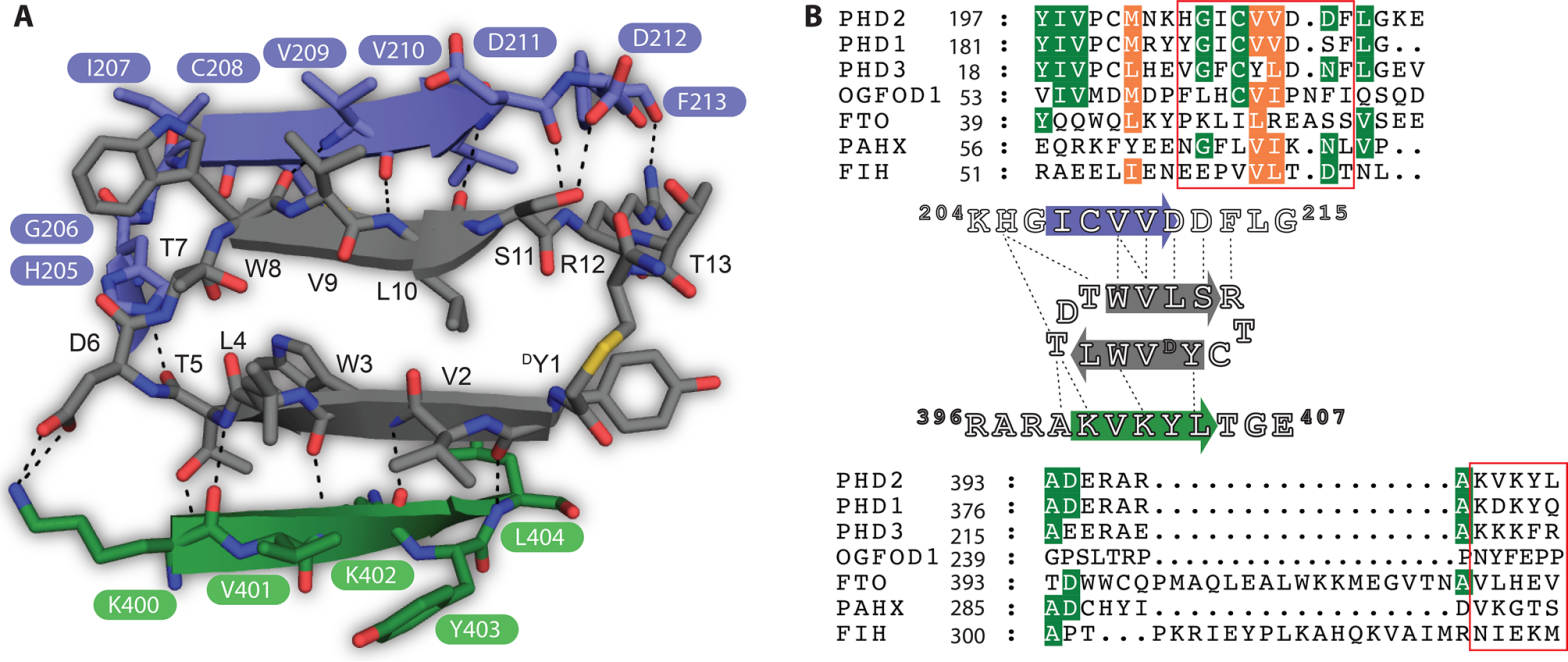
Structure based sequence alignment of 3C-ineracting residues in the PHDs and related oxygenases reveal the basis of its exquisite selectivity for the PHDs. (**A**) By contrast with many other macrocyclic peptide structures with a similar distorted β-sheet fold^31^, 3C has relatively few intramolecular interactions, but makes substantial more intermolecular interactions with cPHD2. 3C residues 8–11 from a parallel β-sheet with PHD2 β1 (aa 205–209) and 3C residues 1–4 form an anti-parallel β-sheet with PHD2 *C*-terminus (aa 400–404). Interestingly, binding of the 3C β-sheet to β1 of PHD2 extends the *N*-terminal side of double stranded β-helix (DSBH) core, which likely stabilizes its overall fold. (**B**) Structure based sequence alignment of 3C interacting regions (boxed black) in PHD1 (PDB: 5V1B), 2 and 3 with OGFOD1 (PDB: 4NHX), FTO (PDB: 4IE5), PAHX (PDB: 2A1X) and FIH (PDB: 1H2K) reveal that the 3C-binding residues are only conserved in PHDs. Dotted lines represent polar interactions of 3C with cPHD2.

**Figure 4:**
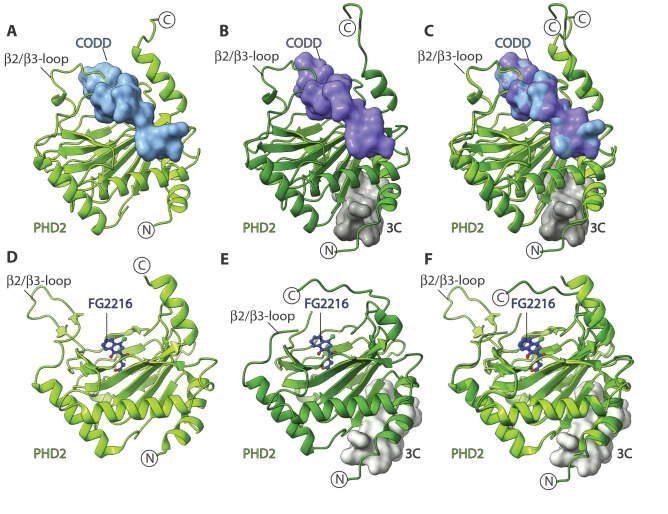
Comparison of PHD2.CODD and PHD2.FG2216 complexes in the presence and absence of 3C. The figure shows views from structures of nPHD2.CODD (PDB ID: 5L9B, **A**), cPHD2.CODD.3C (**B**), superimposed PHD2.CODD complexes (**C**), nPHD2.FG2216 (PDB ID: 4BQX, **D**), cPHD2.FG2216.3C (**E**) and superimposed PHD2.CODD complexes (**F**). Comparison of the PHD2.CODD complexes (**A**–**C**) reveals separate binding sites for 3C and CODD. Binding of 3C does not cause any significant changes in the CODD-binding regions of PHD2 *C*-terminus (up to aa 399) and β2/β3 loop (aa 237–254). Binding of 3C, however, induces conformational changes in the *C*-terminal region of PHD2 (400–405), with implications for crystal packing (see Fig. 5). Comparison of PHD2.FG2216 complexes (*P*6_3_ crystal form) (**D**-**F**) reveals that the β2/β3 loop adopts an open conformation that is stabilised by intermolecular interactions in the crystallographic trimer (see Fig. 1), and that its conformation is likely in part a consequence of the crystal lattice. In the cPHD2.FG2216.3C complex (*P*6_5_ form), the β2/β3 loop is free from crystal packing restraints and appears to adopt a more similar conformation to that observed in the PHD2.CODD complexes, though it is partially disordered.

**Figure 5:**
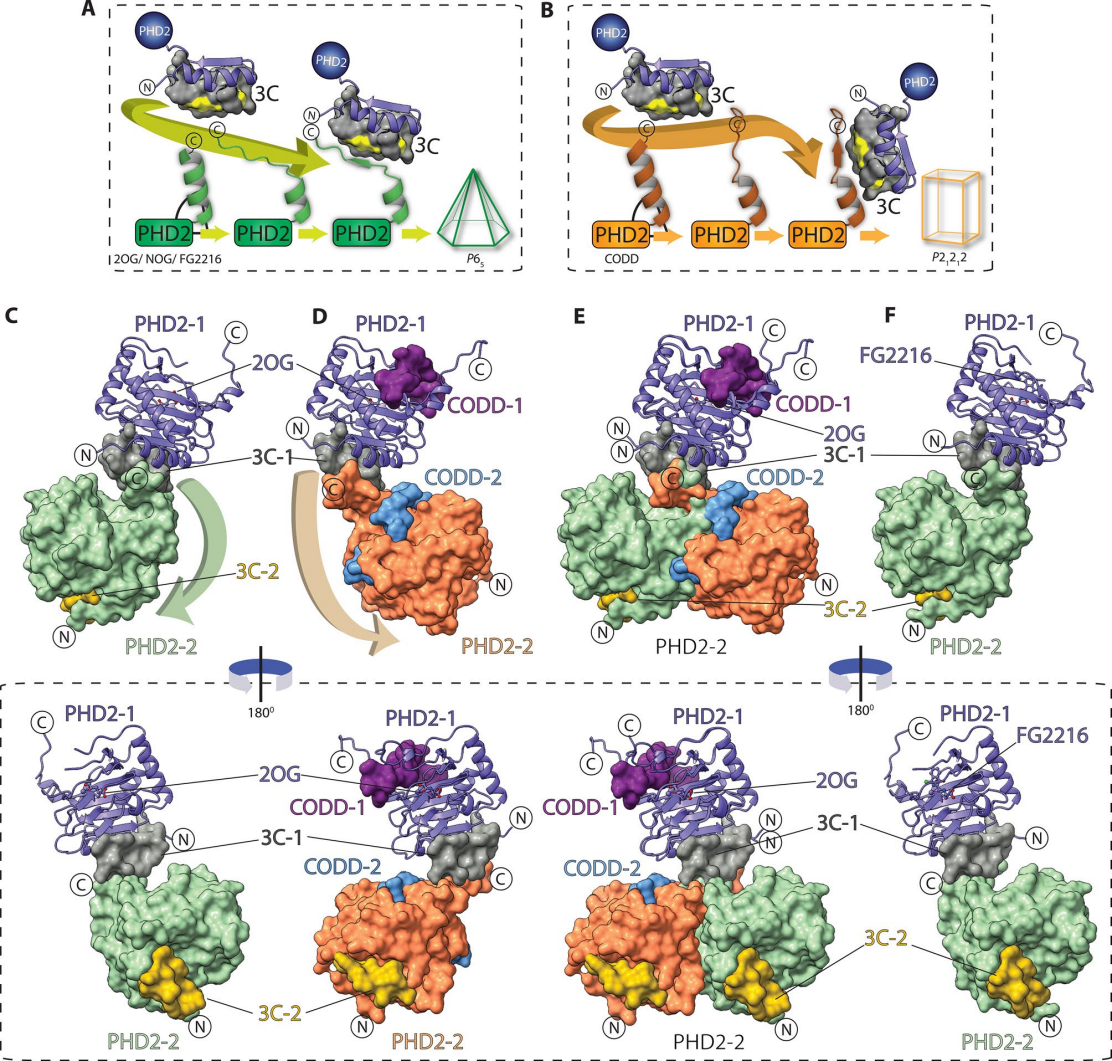
Overview of 3C induced crystal packing in various PHD2 complexes. The figure outlines a proposed scheme of how 3C recognizes different PHD2 *C*-terminal conformations and consequently induces crystallization in different forms. (**A**) and (**B**) show the PHD2 *C*-terminus (green and blue cartoons) as observed in 3C-unbound (starting point) and 3C-bound (end point) states in PHD2 structures with 2OG/NOG/FG2216 (**A**) vs. CODD (**B**). In all 3C-unbound structures, the *C*-terminal residues 393–403 form part of α4; in the cPHD2.3C complexes, α4 only extends to residue 397 before its helicity is broken; this is precisely the point at which 3C interacts with a symmetry-related molecule inducing formation of an extended anti-parallel β-sheet (see Fig. 3). (**C**–**F**) show views from structures of cPHD2.2OG.3C (**C**), cPHD2.2OG.CODD.3C (**D**), superimposed PHD2.2OG.3C and PHD2.2OG.CODD.3C (**E**), and cPHD2.FG2216.3C complexes (**F**) and their corresponding 180° rotated views in a dotted box (bottom panel). Note that when comparing nPHD2.2OG/inhibitor (2OG/PDB: 3OUJ, NOG/PDB: 5L9R, and FG2216/PDB: 4BQX), and nPHD2.CODD (PDB ID: 5L9B) structures, in the latter case, α4 interacts with CODD and moves towards the DSBH core. Thus there is a difference in the spatial relationships between the *C*-termini of the different complexes with respect to their superimposed DSBH cores. 3C can apparently recognize small angular differences in the PHD2 C-terminal region in the 2OG/inhibitor versus CODD complexes and establish intermolecular interactions between the *N*-terminal region of one monomer and the *C*-terminal region of a neighbouring symmetry related molecule. The interaction between 3C and the different PHD2 *C*-terminus conformations results in differences in the relative orientations of PHD2 monomers, which is likely an important factor in determining lattice symmetry. The arrows indicate how binding of 3C to the *N*-terminal region of one monomer (PHD2-1) enables clamping with a symmetry related monomer (PHD2-2).

We successfully crystallized cPHD2 in the presence of a 14-residue NCCP (3C), obtained in a RaPID with five rounds of screening^8^. Multiple crystals were obtained in the presence of 3C and active site binding inhibitors, such as NOG (*N*-oxalylglycine, a close 2OG analogue) and FG2216, in the hexagonal *P*6_5_ crystal form (Figs. 2 and 3). Pleasingly, we also discovered that 3C enables rapid crystallization of cPHD2 in the presence of 2OG (or NOG) and the substrate, HIF-1α CODD (aa 556–574), in the *P*2_1_2_1_2 form (Figs. 4 and 5). The addition of 3C had a dramatic effect on the efficiency of PHD2.substrate complex crystallization; we obtained a large number of cPHD2.3C crystals in complexes with substrate peptides that grew to full size within less than a week, compared to the substantially fewer crystals in > 6 months without 3C^27^.

Consistent with the reported NMR studies^8^, we obtained multiple crystal structures revealing that 3C binds in the region of cPHD2 immediately to the *N*-terminal side of the core distorted double stranded β-helix (DSBH) fold of PHD2 (Fig. 2). Interestingly, within the crystal lattice, 3C ‘slots’ into a tight groove between the non-DSBH β1 of one cPHD2 molecule and the *C*-terminal α4 (that is also involved in the *P*6_3_ crystal packing) of a neighbouring symmetry related cPHD2 molecule (Fig. 2). Binding of 3C at the interface of two symmetry related PHD2 monomers likely promotes contacts productive for crystallization in part via projecting part of the *C*-terminal helix α4 (aa 400–404) away from the active site, in a manner that maintains interactions with substrates that are required for catalysis (Figs. 4 and 5). The *C*-terminal residues 400–404, which form part of the helix α4 in most PHD2 crystal structures without 3C^22,26,27^, are positioned to make anti-parallel H-bonds with 3C (aa 1–4) adopting a β-strand fold in all cPHD2.3C complexes (both with and without CODD) (Fig. 3). Except for the aforementioned *C*-terminal helix α4 residues 400–404, at least in the crystalline state, binding of 3C does not cause any substantial structural changes in the overall fold including of the core DSBH, consistent with the reported NMR studies on the cPHD2.3C complex (Fig. 4).

In the cPHD2.3C complex structures, 3C adopts a rectangular fold comprising two (almost) planar distorted β-strands connected by two type II beta turns with ^D^Y1, D6, W8 and T13 at its four corners (Fig. 3); most of the side chains do not protrude extensively (Fig. S4). To date, there is only a single reported structure of a 2OG-dependent oxygenase, i.e. the JmjC histone demethylase KDM4A in complex with a CP, where the cyclic peptide binds at the substrate interacting site and is inhibitory^31^. By comparison with the KDM4A.CP complex structure, 3C forms fewer intramolecular, but substantially more intermolecular interactions with cPHD2 (Fig. S5). 3C forms extensive backbone-to-backbone interactions with cPHD2 β1 (aa 204–214) and three residues located at the *N*-terminus (187–189) within the same cPHD2 monomer and six residues (399–404) located at the *C*-terminus of a symmetry related cPHD2 molecule (Fig. 3 and Fig. S5). In addition, there are backbone to side-chain polar interactions between L188_PHD2_ and F213_PHD2_ with R12_3C_, and A399_PHD2_ with T5_3C_, as well as side-chain to side-chain electrostatic/H-bond interactions of D212_PHD2_ with T13_3C_ and S11_3C_, and of K186_PHD2_ with Y^D^1_3C_ (Fig. 3). Although the 3C interacting PHD2 residues are well-conserved in all three human PHDs, they are different in other 2OG-oxygenases including the ribosomal prolyl hydroxylase (OGFOD1, Z = 17.4)^36^, nucleic acid demethylases (e.g., FTO, Z = 14.4)^37^, phytanoyl-CoA dioxygenase (PAHX, Z = 13.3)^38^ and factor inhibiting HIF (FIH, Z = 10.8)^39^, providing a structural basis for the high selectivity of 3C and related NCCPs for the PHDs (Fig. 3)^8^.

PHD catalysis involves coordinated movements, including of two flexible regions comprising a dynamic β2/β3 loop and the *C*-terminal helix region (including α4) (Fig. 4) that are directly involved in substrate binding^26,27,40^. Although binding of 3C induces structural changes in cPHD2 *C*-terminal residues (400–404) that form part of the *C*-terminal helix (α4), 3C binding allows protein–protein interactions between the cPHD2 *C*-terminus and the HIF-α substrate, consistent with the catalytic activity observed in the presence of 3C (Fig. 5). Comparison of HIF-α ODD substrate complex structures, i.e. nPHD2.2OG.CODD (PDB : 5L9B)^26^ and cPHD2.2OG.CODD.3C, reveals that 3C does not cause any substantial conformational changes in the β2/β3 loop which folds to enclose the substrate (Fig. 4), nor in any of the identified (by crystallography or NMR^26,27^) substrate binding or active site regions, an observation consistent with our biochemical observations^8^.

## Discussion

We initially employed extensive screening of crystallization conditions to obtain PHD2 crystals. This work led us and others^21–24^ to the finding that nPHD2 readily crystalizes in the presence of particular heteroaromatic inhibitors, the side chain of which occupies the 2OG co-substrate binding site (the ‘*P*6_3_’ crystal form). The *P*6_3_ form is, however, not amenable to different types of ligand/substrate complex crystallization. With the aim of altering PHD2 crystal packing to enable efficient and robust generation of enzyme-ligand/substrate complexes, we screened for different types of active site binding ligands and employed surface methylation of lysine-residues.

Although reductive *N*^ε^-methylation of nPHD2 lysines did not enable us to obtain a different crystal packing other than the *P*6_3_ form, *N*^ε^-methylation appears to improve the structural order/conformational stability for some of the flexible regions of nPHD2, suggesting it may be useful in crystallizing other oxygenases, including full length PHD constructs. In general, reductive alkylations of proteins are more likely complete or successful when target lysines are solvent exposed with relatively high accessible surface areas (ASA)^2,29^, as was the case with nPHD2.

Unexpectedly, the work with small molecule ligands led to the observation that the ability to induce formation of the homotrimeic crystal form does not correlate with either their active site ligand binding efficiency or inhibitory potency. This observation is notable because many structural biology approaches involving crystallography are aimed at identifying and exploiting tight binding ligands for the isolated macromolecule^3^. It is presently impractical to exhaustively screen very large numbers of combinations of crystallization conditions and weakly binding ligands; however, employing focused relevant compound sets, e.g. of diverse 2OG analogues in the case of 2OG oxygenases, and a sparse matrix method using ligand grid screens as we employed in this work may have wider applications. Crystallization of the nPHD2 in complex with weakly binding succinimide derivatives (SCAs) occurs under the physicochemical conditions established for nPHD2.FG2216 type inhibitor crystallization^21,22^. Compounds from both the FG2216 and SCA series enable nPHD2 crystallization with approximately equal efficiency, yet FG2216 (and related compounds) are significantly more potent inhibitors than the SCAs under the tested assay conditions. Nonetheless, the variable degrees of inhibition of different 2OG-oxygenases by the SCAs and related compounds (Fig. S1) opens up new possibilities for selective inhibitor design, e.g. by using knowledge of the active site, it may be possible to improve binding of the SCA series and induce PHD2 oligomerization as a means of inhibition.

**Figure 6:**
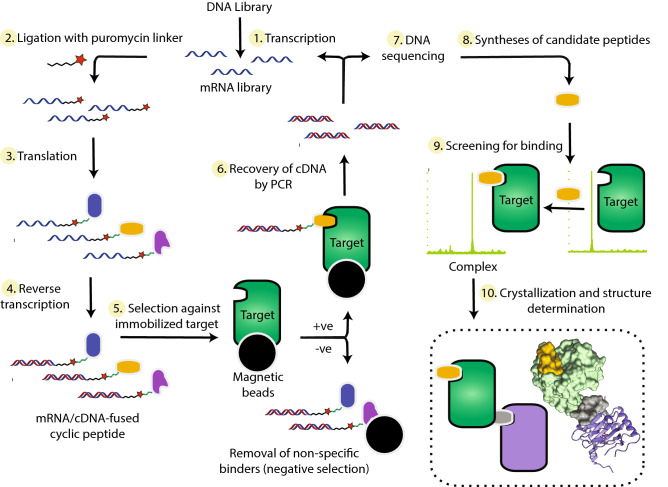
Overview of the RaPID selection procedure coupled to protein structure determination. See references^8,9^ for more details. The starting DNA library is transcribed into an mRNA pool which is ligated with puromycin-derivatized oligonucleotides, then used as templates for in vitro translation. This translation reaction mixture contains 19 proteinogenic amino acids (excepting methionine) and is supplemented with an initiator tRNA acylated with chloroacetyl-d-tyrosine. The peptide is cyclised by intramolecular S_N_2 reaction between the chloroacetyl group and a *C*-terminal cysteine. The puromycin covalently links the coding mRNA strand to the corresponding translated CPs, so reverse transcription generates mRNA/cDNA-fused CPs. The mixture is incubated with magnetic beads to select for CPs binding to the immobilized target, then washed, before the cDNA associated with bound CPs is PCR amplified and sequenced. The peptides thus identified are synthesized by standard solid-phase synthesis, incubated with the target protein, and tested by MS-based screening (or other binding assay) for complex formation (kinetic assays may also be performed). Co-crystallization experiments involve mixing CPs and target protein typically at a 1–1.1:1 molar ratio, respectively. Alternatively, the complex can be purified by size exclusion chromatography. RaPID screens could also be performed on enzyme–substrate/-inhibitor complexes to promote identification of non-competitive CPs (NCCPs). Owing to their typically high stability, CPs are well-tolerated for screening a broad range of crystallization conditions with extremes of pH, temperature, ionic strength, ligands/additives, and precipitants. Thus, crystallization conditions screened are limited by the stability of the target protein, not that of the CP crystallization agent, which is often an issue with antibodies/‘crystallization chaperones’^5^.

The above procedures, i.e. use of orthosterically binding ligands/reduction of surface entropy, did not lead to desirable new PHD2 crystal forms, leading us to explore unconventional methods for crystallization. Following screening for cyclic peptides (CPs) binding to PHD2 via a modified mRNA display methodology (Fig. 6), we identified a 14-mer cyclic peptide thioether (3C) that promotes crystallization of the catalytic domain of PHD2, whilst still enabling a catalytically productive substrate binding mode. 3C is a powerful tool, because it enables efficient crystallization of PHD2 in complexes with PHD inhibitors, including those that are in clinical trials, but potentially also for conducting more detailed structural analyses of catalytic intermediates, e.g. by time-resolved crystallography. Given the efficiency and cost-effectiveness of the RaPID methodology (e.g. compared to classical high throughput small-molecule screening) and the availability of peptides on scales suitable for biophysical analyses via solid phase synthesis, it is possible that the method will have general applicability in crystallization (Fig. 6).

In our case, the method yielded an NCCP that does not interfere with substrate binding; this is likely in part because the active site pocket of PHD2 can be obscured by dynamic loop conformations involved in induced fit during catalysis^26^, thus biasing the RaPID screen to identifying peptides binding elsewhere. In some cases, it may be productive to protect or block an active site/pocket (e.g. by a tight binding ligand) or other potential interaction sites (e.g. protein-nucleic acid/protein–protein interaction motifs) during the mRNA display screening process in order to identify surface binding NCCPs for promoting crystallization.

The human PHDs catalyse hydroxylation of prolyl-residues within the *N*/*C* terminal ODDs of HIF-1/2/3α and are negative regulators of the transcriptionally regulated hypoxic response^11,12^. In addition, there are reports that the PHDs catalyse prolyl hydroxylation of non-HIF-α substrates in cells^41^, though these reports need to be validated^42^. Except for HIF-1α NODD and CODD^26,27^, there is no structural information available on how PHDs catalyse hydroxylation of different HIF isoforms or non-HIF substrates and how they achieve selectivity in cells. Our work demonstrates the potential for NCCPs for structural analyses of PHD complexes, not only with inhibitors, but with different HIF ODDs/potential non-HIF substrates, which have otherwise been difficult to achieve.

The longstanding challenge of efficiently crystallising proteins has motivated efforts to develop innovative methods that induce ordered oligomerization. Use of highly soluble proteins as fusions has enabled determination of structures of many classes of proteins. For example, use of T4 lysozyme to replace part of the third intracellular loops or the *N*-termini of G protein-coupled receptors (GPCRs) has enabled determination of the structures of several GPCR complexes^4,43,44^. However, in many cases (including GPCRs), the production of such fusion proteins often leads to loss of activity or a failure to yield well diffracting crystals^45^. There are also many examples of using small molecule additives and auxiliary proteins, sometimes termed ‘crystallization chaperones’, including antibodies, nanobodies/FAB fragments, to aid crystallization^5,46,47^. Nanobodies have elicited interest in chaperoning protein crystallization due to their ability to reduce conformational heterogeneity and shield unproductive surfaces from solvents, whilst extending crystallographically productive surfaces to form crystal contacts^4,47^. However, generating antibodies/nanobodies and characterizing their complexes with protein of interest is time-consuming and can lead to structures that are not biologically representative^46,47^.

Although our results involve a specific case, together with other studies on CPs^9,10^, they suggest that use of readily synthesised non-competitive peptides (NCCPs) that can bind at the intermolecular interfaces between proteins and induce crystallogenesis are worthy of further investigation as a more general method to aid in protein/macromolecular crystallization. Although there is effort in initially setting up the RaPID method (Fig. 6), once established, it is robust, cost-effective (especially compared to operation of large small molecule libraries) and is easy to operate^9^. CP assisted crystallization has the potential advantage over the use of nanobodies or other recombinant crystallization ‘chaperones’ that the CPs are relatively small, being typically < 20 residues, compared to a single immunoglobulin domain of ∼125 residues. The use of CPs is thus likely to increase the chance of preserving native folds compared to crystallization chaperones. Compared to some antibody based methods, CP generation does not need for animal immunization/hybridoma technology. Once identified, the CPs can be easily synthesized or purchased, without requiring any specialized expression system. By contrast with proteins prepared by translation, since the CPs are prepared by synthesis, non-proteinogenic/unnatural residues can also be readily incorporated into them. CPs thus are stable, low mass, cost-effective, and tight binding molecules (*K*_D_ values in the range of nM to pM)^9^, which are suited for crystallization screening in a wide range of physicochemical conditions.

## Materials and methods

Recombinant PHD2 proteins were produced in *E. coli* and purified by metal affinity and size exclusion chromatography as reported^26^. The in vitro selection of CPs binding to biotinylated His_6_-PHD2 was carried out using RaPID methodology as reported^8^. Peptides were produced by standard Fmoc-solid phase peptide synthesis, all with an amidated *C*-terminus and a chloroacetylated *N*-terminus. Peptides were cleaved from the resin with a TFA-based cleavage mixture, cyclised to give a thioether link, then purified by HPLC as reported^8^. Assays comprised incubation with Fe(II)/2OG/substrate(s) followed by MS and/or NMR analyses as reported^26^. cPHD2.3C complex crystals were grown by vapour diffusion at 22 °C in 300 nL sitting drops with 2:1 or 1:1 or 1:2 ratio of sample (1.0 mM cPHD2, 1.5 mM MnCl_2_, 2.0 mM 2OG/NOG/FG2216, 1.0 mM 3C, with/without 2.0 mM CODD) to well solution (19–20% w/v polyethylene glycol 3350, 0.3 M magnesium formate, 2 mM MnCl_2_) and cryo-cooled in N_2_(l). Details of nPHD2.SCA complex crystallization are in Supplementary Methods. Data were collected at the Diamond Light Source (DLS) and the European Synchrotron Radiation Facility (ESRF) MX beamlines (Tables S1 and S2). The structures were solved by molecular replacement using PDB ID: 5L9B, 4BQX, and 5L9R as initial models. See Supplementary Methods for details.

### Statistical analysis

Endpoint assay results are the mean of three independent experiments with error bars representing the s.e.m. For kinetic measurements, each experiment was carried out (at least) in triplicate (n = 3–5).

## Supplementary information

Supplementary Information

## Data Availability

GenBank accession codes for the sequences mentioned in this article are as follows: Q9GZT9 (EGLN1_HUMAN); Q16665 (HIF1A_HUMAN). Atomic coordinates and structure factors for the crystal structures of nPHD2.**4a** (PDB: 6YVX), nPHD2.**7f.** (PDB: 6YVW), nPHD2.**30a** (PDB: 6YVZ), nPHD2-Me.**4a** (PDB: 6YW0), cPHD2.2OG.3C (PDB: 6YW1), cPHD2.FG2216.3C (PDB: 6YW2), cPHD2.NOG.3C (PDB: 6YW4) and cPHD2.NOG.3C.CODD (PDB: 6YW3), and are deposited in the protein databank (wwPDB) and will be released on acceptance. Additional data supporting the findings of this study are available from the corresponding authors on request.
