# Supplementary material for: Use of cyclic peptides to induce crystallization: case study with prolyl hydroxylase domain 2
Source: Sci Rep. 2020 Dec 15;10:21964. doi: 10.1038/s41598-020-76307-8 (PMC7738489; doi:10.1038/s41598-020-76307-8)
Supplement: Supplementary file 1 — Supplementary Information [file 41598_2020_76307_MOESM1_ESM.docx]

**Supplementary Information: Use of Cyclic Peptides to Induce Crystallization – Case Study with Prolyl Hydroxylase Domain 2**

Rasheduzzaman Chowdhury, Martine I. Abboud, Tom E. McAllister, Biswadip Banerji, Bhaskar Bhushan, John L. Sorensen, Akane Kawamura, and Christopher J. Schofield*

Chemistry Research Laboratory, Department of Chemistry, University of Oxford, Oxford OX1 3TA, United Kingdom.

email: christopher.schofield@chem.ox.ac.uk.

**Supplementary Methods**

Materials

PHD2 production

Reductive lysine methylation

Solid-phase synthesis of SCAPs

Endpoint assays

Mass spectrometry

NMR Spectroscopy

Crystallography

General Synthetic procedures

**Supplementary Figures**

Figure S1. Inhibition of 2OG-oxygenases by succinamic acid derivatives (SCAs).

Figure S2. NMR and MS analysis of PHD2.SCA complexes

Figure S3. PHD2 lysine-residue methylation

Figure S4. The 3C peptide conformation as observed in the cPHD2.3C complexes.

Figure S5. Polar interactions between the cyclic peptide 3C and cPHD2 N/C-terminal residues.

**Supplementary Tables**

Table S1. Data collection and refinement statistics of the nPHD2.SCA complexes.

Table S2. Data collection and refinement statistics of the cPHD2.3C complexes.

**Supplementary References**

**Supplementary Methods**

**Materials**. Chemicals were from Sigma-Aldrich, except where stated. MALDI matrix buffers and calibrants were from LaserBioLabs. All peptide substrates, including HIF-1α CODD_556-574_ (DLDLEMLAPYIPMDDDFQL) were from GLS Biochem (China) and were prepared as C-terminal amides. Flash chromatography was performed using silica gel (0.125-0.25 mm, 60-120 mesh) as the stationary phase. Thin layer chromatography (TLC) was performed using aluminium plates pre-coated with silica gel (Merck silica gel 60 F_254_ 1.05554), which were visualized by UV (λ_max_ = 254nm), and/or by staining with iodine or KMnO_4_ in solution, followed by heating. ^1^H-NMR (500 MHz) and ^13^C-NMR (125.8 MHz) spectra were recorded using Brüker DRX 500 AMX 500 spectrometers at ambient temperature. Coupling constants (*J*) are ± 0.5 Hz. Chemical shifts (δ) are in parts per million (ppm) relative to the residual non-deuterated solvent signal. High-resolution mass spectra were recorded using a VG Autospec spectrometer by chemical ionization or on a Micromass LCT electrospray ionization mass spectrometer operating at a resolution of 5000 full width half height.

**PHD2 production.** PHD2_181-426_ (nPHD2) construct was prepared as reported using the pET-28a vector[^1^](#_ENREF_1). The PHD2_181-407_ (cPHD2) variant was prepared using the nPHD2/pET-28a construct by site-directed mutagenesis (Stratagene). All constructs were verified by DNA sequencing. In brief, recombinant nPHD2 and cPHD2 proteins were produced in *Escherichia coli* BL21(DE3) cells by induction with 0.5 mM IPTG for 3-4 hours at 37°C.[^1^](#_ENREF_1) Cells were harvested, freeze-thawed and lysed in 20 mM Tris·HCl, pH 7.0, 0.5 M NaCl by sonication. nPHD2 and cPHD2 were purified by Ni^2+^ affinity (tetracarboxymethyl ethylenediamine, TED) and size exclusion chromatography (SEC)[^1^](#_ENREF_1)^,^[^2^](#_ENREF_2). Protein purity (>90%) was assessed by SDS-PAGE; protein identities were confirmed by MS analyses under non-denaturing and denaturing conditions.

**Reductive lysine methylation**. Protein lysine methylation was performed as described[^3^](#_ENREF_3) using nPHD2 (0.5 mg/mL) in 50 mM HEPES-Na pH 7.5 buffer. In brief, 20 μL of freshly prepared 1 M dimethylamine-borane complex and 40 μL of 1 M aqueous formaldehyde were added per mL of nPHD2 solution; the resultant solution was gently mixed and incubated at 4°C. After 2 hours, a further 20 μL of 1M dimethylamine-borane complex solution and 40 μL of the formaldehyde solution were added and the incubation was continued for another 2 hours. Following a final addition of 10 μL1M dimethylamine-borane complex, the reaction was incubated overnight at 4°C. The reaction mixture was then centrifuged to remove any unwanted precipitation before being purified by SEC. Reductive methylation of nPHD2 yielded two products (nPHD2Me-I and nPHD2Me-II) that were well-resolved by SEC as described[^1^](#_ENREF_1)^,^[^2^](#_ENREF_2). The retention times of the two peaks observed suggests that the methylation product, nPHD2Me-I, eluting near the column void volume is likely aggregated. Peak fractions corresponding to nPHD2Me-II (nPHD2-Me in the main text) were pooled and concentrated for further studies.

**Solid-phase synthesis of SCAPs**. The cyclic peptide, 3C, was produced (with a C-terminal amide) by standard solid-phase peptide synthesis as described[^4^](#_ENREF_4).

**Endpoint assays**. A time-resolved fluorescence resonance energy transfer (TR-FRET) assay was employed for inhibition studies using the reported protocol[^5^](#_ENREF_5). 2OG turnover was measured by assaying [^14^C]-CO_2_ production[^1^](#_ENREF_1). Cell-based assays were performed as described[^6^](#_ENREF_6).

**Mass spectrometry**. Mass spectrometry under non-denaturing conditions was performed as described[^1^](#_ENREF_1)^,^[^7^](#_ENREF_7). For LC-MS/MS analyses, samples were buffer exchanged, then digested ‘in solution’ with trypsin (Promega) in 20 mM ammonium bicarbonate; incubations were carried out for ~16 hours at 37 ºC. An Agilent 1100 capillary LC system (Palo Alto, CA) was used for separation; 8 μL of the digest solution was injected onto a 5 μm Phenomenex Jupiter™ capillary LC column (150 × 0.5 mm). The fragment peptides were resolved with a linear gradient of 5-95% (*v/v*) solvent B for 50 minutes, followed by a second gradient of 95-5% for 40 minutes at a flow rate of 15 μl·min^-1^. Mobile phase solvent A was 0.1% (*v/v*) aqueous formic acid and solvent B comprised 0.1% (*v/v*) aqueous formic acid in 90% (*v/v*) acetonitrile. The LC column eluate was analysed from 0 to 35 minutes using positive ion electrospray MS employing a Waters (Milford, MA) Q-Tof Micromass spectrometer.

**NMR Spectroscopy**. Nuclear Magnetic Resonance (NMR) spectra were recorded with a Bruker AVIII 600 MHz NMR spectrometer equipped with a BB-^19^F/^1^H Prodigy N_2_ cryoprobe using 3 mm diameter NMR tubes (Norell)[^8-11^](#_ENREF_8); Data were processed with the Bruker 3.1 software.

*^1^H CPMG NMR.* Typical experimental parameters for Carr-Purcell-Meiboom-Gill (CPMG) NMR spectroscopy were: total echo time, 40 ms; relaxation delay, 2 s; and number of transients, 264. The PROJECT-CPMG sequence (90°x−[τ−180°y−τ−90°y−τ−180°y−τ]n−acq) was applied. Water suppression was achieved by pre-saturation. Prior to Fourier transformation, the data were multiplied with an exponential function with 2 Hz line broadening.

*wLOGSY NMR experiments*. water-Ligand Observed Gradient SpectroscopY (wLOGSY) experiments[^10^](#_ENREF_10)^,^[^11^](#_ENREF_11) were conducted using with typical experimental parameters as follows: mixing time, 1 s; relaxation delay, 2 s; number of transients, 256. Solvent excitation was achieved using a 16 ms 180° selective rectangular shape pulse with 1000 points (Squa100.1000) set at the H_2_O frequency. Water suppression was achieved by a 2 ms Sin pulse (Sinc1.1000) pulse at the H_2_O frequency.

**Crystallography**. Initial high-throughput screens investigated in attempts to crystallize both nPHD2 and cPHD2 proteins with variable ligands included: Crystal Screen, Crystal Screen 2, Index, PEG 6000 Grid Screen, Ammonium Sulfate Grid Screen, Salt-Rx and PEG/Ion (Hampton Research), and the Wizard I and II (Emerald BioSystems). Crystals of nPHD2.Fe(II).**4a**/ **7f** (~20 mg·mL^-1^ protein + 1 mM FeSO_4_ + 2 mM compound) were grown by hanging drop vapour diffusion at 20°C under near anaerobic (*P*O_2_ <0.1 ppm)[^12^](#_ENREF_12) conditions, whilst crystals of nPHD2.Mn(II).**30a** (~20 mg·mL^-1^ protein + 1 mM MnCl_2_ + 2 mM **30a**) were grown at ambient temperature and under aerobic conditions. The crystallization buffer used contained: 1.6–2.0 M (NH_4_)_2_SO_4_, 2–8% (v/v) aqueous dioxane, 0.1 M MES-Na pH 6.5, and 1 mM FeSO_4_/ MnCl_2_. Stock solutions of SCAs were prepared using 100 % DMSO to a final concentration of 0.1 M. nPHD2-Me.Fe(II).**4a** (~18 mg·mL^-1^ protein + 1mM FeSO_4_ + 2 mM **4a**) crystallized in well conditions containing 1.7 % (v/v) aqueous polyethylene glycol 400 (v/v), 15 % glycerol (v/v), 1.7 M ammonium sulphate and 0.085 M HEPES-Na pH 7.5. cPHD2.3C complex crystals were grown at 20 °C in 300 nl sitting drops with 2:1 or 1:1 or 1:2 ratio of sample (1.0 mM cPHD2, 1.5 mM MnCl_2_, 2.0 mM 2OG/ NOG/ FG2216, 1.0 mM 3C, with/ without 2.0 mM CODD) to well solution (19-20% w/v polyethylene glycol 3350, 0.3 M magnesium formate, 2 mM MnCl_2_) Crystals were transferred directly into cryo-solution containing crystallization buffers and 25% glycerol and frozen in liquid N_2_.

As described in Supplementary Tables S1-S2, data were collected at 100K using synchrotron radiation at the Diamond Light Source (DLS) and the European Synchrotron Radiation Facility (ESRF) beamlines unless otherwise mentioned. Data were processed as outlined in Supplementary Tables S1-S2. Structures were solved by molecular replacement using PHASER[^13^](#_ENREF_13) (search model PDB ID 4BQX or 5L9R)[^2^](#_ENREF_2)^,^[^14^](#_ENREF_14) and refined by alternative cycles of PHENIX[^15^](#_ENREF_15), CNS[^16^](#_ENREF_16) and BUSTER[^17^](#_ENREF_17) using the maximum-likelihood function and bulk-solvent modelling. The initial model used for nPHD2-Me did not include any methylation of lysine residues. Iterative cycles of model building in COOT[^18^](#_ENREF_18) and refinement proceeded until the R_cryst_/R_free_ values converged. Final rounds of refinement were performed by PHENIX[^15^](#_ENREF_15). MOLPROBITY[^19^](#_ENREF_19) was used to monitor the geometric quality of the models between refinement cycles and identify poorly modelled areas needing attention. Water molecules were added to peaks >1.2σ in 2*F*_o_ – *F*_c_ electron density maps that were within hydrogen bonding distance to protein with reasonable hydrogen bonding geometry and were refined by PHENIX[^15^](#_ENREF_15).

**General Synthetic procedures.**

*Amide Coupling-1 (Schemes 1, 2, 3, 4, 7).* To a solution of the relevant amine (isoquinoline-3-amines, **1** / substituted-2-amino pyridines, **5** / benzothiazole-2-amine, **8** / hydrazides, **11** or **15** / quinoline-2-amine, **21**/) (1eq) in CH_3_CN, was added pyridine (1.2 equiv.) followed by the relevant acylchloride **2**, (1.1eq) at 0^o^C. The reaction mixture was warmed to room temperature for 30 min, then stirred under ambient temperature for 1 hour. After this period, the solvent was removed in vacuo and the residue was loaded onto a flash chromatography column (silica gel; EtOAc: hexanes) to afford the purified amides **3**, **6**, **9**, **12**, **16** and **22** in moderate to good yields.

*Amide Coupling-2 (Schemes 4, 5, 6, 8).* To a solution of quinoline-2-carboxylic acid, **14a** or **24**/ naphthaline-2-carboxylic acid, **14b**/ pyridine-2-carboxylic acid/ isoquinoline-3-carboxylic acid, **18** (1 equiv.) in CH_3_CN was added Et_3_N (3.0 eq), followed by PyBOP^®^ (1.2 equiv.); the reaction mixture was stirred at ambient temperature for 15 mins. After this period, amine-ester hydrochloride, **2** / hydrazides, **11** (1.5 equiv.) were added to the reaction mixture which was then stirred for 5 hrs at room temperature. The solvent was then removed in vacuo, and the residue was dissolved in EtOAc, washed with water (2 × 10 mL) and then brine (1 × 10 mL). The combined organic layers were dried (MgSO_4_), filtered, then concentrated in vacuo to afford a residue which was purified (flash column chromatography; silica gel; EtOAc: hexanes) to afford the purified esters **15**, **16**, **19**, **25** and **27** in moderate 55-65 % yields (Scheme 1).

*Saponification.* To a solution of ester (1eq) in a mixture of THF and H_2_O (4:1) at room temperature was added LiOH (1.2 eq). After the 2-4 hrs (upon completion of reaction as observed by TLC), the solvent was removed in vacuo and the reaction mixture was diluted with water, washed with EtOAc (2 × 10 mL). The aqueous layer was then acidified with 1N HCl to pH ~4 to afford a white precipitate, which was in some cases collected by filtration washed with water, then dried to afford the desired acid as a white solid. In other cases, the solid was extracted into EtOAc, washed with water and dried (MgSO_4_), filtered and concentrated under reduced pressure to afford the corresponding acids **4**, **7**, **10**, **13**, **15**, **20**, **23**, **26** and **28** in 90-95% yields.

**Legends to Scheme 1:** (a) **1** (1 eq), pyridine (1.2 eq), **2** (1.1 eq), MeCN, 0^o^C, 1 h, 55-65%; (b) **3** (1 eq), LiOH (1.2 eq), THF: H_2_O (4:1, 10 mL), RT, 2 h, 90-95%

**Spectral characterization of the compounds**

**3a**: δ_H_ (500MHz; CDCl_3_) 9.07 (1 H, brs), 9.0 (1 H, s), 8.63 (1 H, s), 7.93 (1 H, d, *J* = 9), 7.82 (1H, d, *J* = 8.8), 7.69 (1 H, t, *J* = 7.3), 7.52 (1 H, dd, *J* = 8.2, 7.2), 3.73 (3 H, s), 2.85-2.79 (4 H, m); δ_C_ (125MHz; CDCl_3_) 176.5, 170, 149.5, 145, 138, 135, 132, 128, 127.5, 126, 108.5, 52, 32, 29; m/z (EI) 259.1083 (M^+^ + H^+^ for C_14_H_15_N_2_O_3_^+^ requires 259.1076); Melting point: 148^o^C.

**4a:** δ_H_ (500MHz; DMSO-d_6_) 12.5 (1 H, br s), 10.5 (1 H, br s), 9.14 (1 H, s), 8.46 (1 H, s), 8.05 (1H, d, *J* = 8.5), 7.87 (1 H, d, *J* = 8.6), 7.70 (1 H, dd, *J* = 8.5, 7), 7.52 (1 H, dd, *J* = 8.2, 7), 2.71-2.68 (2 H, m), 2.57-2.54 (2 H, m)HH; δ_C_ (125MHz; DMSO-d_6_) 174, 171, 151, 147, 137, 131, 127.5, 126, 125.5, 125, 31, 28.5; m/z (EI) 243.0770 (M^+^ - H^+^ for C_13_H_11_N_2_O_3_^+^ requires 243.0772); Melting points: 202^o^C.

**3b:** δ_H_ (500MHz; CDCl_3_) 8.5 (1 H, br s), 8.2 (1 H, dd, *J* = 8, 8.8), 7.79 (1 H, d, *J* 8.5), 7.69 (1 H, dd, *J* = 8.2, 8.5), 7.58-7.55 (1H, m), 3.74 (3 H, s), 2.81-2.75 (4 H, m); δ_C_ (125MHz; CDCl_3_) 173, 169.5, 144.5, 142.5, 139.5, 131.5, 128.5, 127.5, 127, 126.5, 108, 52, 32, 28.5; m/z (EI) 359.0002 (M^+^ + Na^+^ for C_14_H_13_N_2_O_3_Br^+^ requires 359.000), Melting point: 198^o^C. C_13_H_11_N_2_O_3_^+^ requires 243.0774); Melting points: 191^o^C.

**4b:** δ_H_ (500MHz; DMSO-d_6_) 11 (1 H, s), 8.50 (1 H, s), 8.11 (1 H, d, *J* 8.5), 7.96 (1 H, d, *J* = 8.5), 7.79 (1H, dd, *J* = 8.5, 8.2), 7.67 (1 H, dd, *J* = 8.5, 8.2), 2,70-2.67 (2 H, m), 2.57-2.54 (2 H, m); δ_C_ (125MHz; DMSO-d_6_) 174, 171.5, 146.5, 142.5, 139.5, 132, 128, 127.5, 127, 125.5, 107.5, 31, 28.5; m/z (EI) 320.9875 (M^+^ - H^+^ for C_13_H_10_N_2_O_3_Br^+^ requires 320.9880); Melting points: 190^o^C.

**3c:** δ_H_ (500MHz; CDCl_3_) 8.98 (1 H, s), 8.6 (1 H, s), 8.25 (1 H, br s), 7.90 (1 H, d, *J =* 9), 7.82 (1 H, d, *J* = 9.4), 7.66 (1H, dd, *J* = 8.5, 8.2), 7.5 (1 H, dd, *J* = 8.2, 7), 3.70 (3 H, s), 2.56-2.53 (2 H, m), 2.50-2.48 (2 H, m), 2.15-2.09 (2 H, m); δ_C_ (125MHz; CDCl_3_) 173.5, 170.5, 150.5, 146, 138, 130.5, 127.5, 127, 126.5, 125.5, 108, 51.5, 36.5, 33, 20.5; m/z (EI) 295.1053 (M^+^ + Na^+^ for C_15_H_16_N_2_O_3_Na^+^ requires 295.11).

**4c**: δ_H_ (500MHz; DMSO-d_6_) 12 (1 H, br s), 10.5 (1 H, s), 9.1 (1 H, s), 8.5 (1 H, s), 8.04 (1H, d, J = 8.8), 7.89 (1 H, d, *J* = 8.8), 7.71 (1 H, dd, *J* = 8.2, 8.5), 7.53 (1 H, dd, *J* = 7.9, 8.2), 2.37 (2 H, m), 2.31-2.23 (2 H, m), 1.86-1.82 (2 H, m); δ_C_ (125MHz; DMSO-d_6_) 174, 171.5, 151, 147, 137, 131, 127.5, 126.5, 126, 125.5, 106.5, 35, 33, 20.5; m/z (EI) 259.1077 (M^+^ + H^+^ for C_14_H_15_N_2_O_3_^+^ requires 259.11).

**Legends to Scheme 2:** (a) **5** (1 eq), pyridine (1.2 eq), **2** (1.1 eq), MeCN, 0^o^C, 1 h, 65-75%; (b) **6** (1 eq), LiOH (1.2 eq), THF: H_2_O (4:1, 10 mL), RT, 2-4 h, 90-95%

**Spectral Characterization of the compounds:**

**6a:** δ_H_ (500MHz; CDCl_3_) 8.73 (1 H, br s), 8.27 (1 H, d, *J* = 7.5), 8.22 (1 H, d, *J* = 9.2), 7.73 (1 H, d, *J* = 8.5, 7.5), 7.06 (1H, d, *J* = 8.5, 5), 3.72 (3 H, s), 2.76 (4 H, m); δ_C_ (125MHz; CDCl_3_) 173, 170, 151, 147, 138.5, 119.5, 114, 52, 32, 29; m/z (EI) 209.0926 (M^+^ + H^+^ for C_10_H­_13_N_2_O_3_^+^ requires 209.0925).

**6b:** δ_H_ (500MHz; DMSO-d_6_) 11 (1 H, br s), 8.03 (1 H, d, *J* = 8.5), 7.83 (1 H, dd, *J =* 8.2, 7.9), 7.19 (1 H, d, *J* = 7.9), 3.60 (3H, s), 2.70-2.67 (2 H, m), 2.61-2.58 (2 H, m); δ_C_ (125MHz; DMSO-d_6_) 172.5, 171, 152, 148, 141.5, 119, 111.5, 51.5, 30.5, 28; m/z (EI) 265.0350 (M^+^ + Na^+^ for C_10_H_11_ClN_2_O_3_Na^+^ requires 265.04); Melting points: 130^o^C.

**7b**: δ_H_ (500MHz; DMSO-d_6_) 12 (1 H, s), 10.8 (1 H, br s), 8.04 (1 H, d, *J* 8.5), 7.83 (1 H, dd, *J* = 8.2, 8.5), 718 (1H, d, *J* = 7.6), 2.64-2.62 (4 H, m); δ_C_ (125MHz; DMSO-d_6_) 173.5, 171.5, 152, 148, 141.5, 118.5, 112, 31, 28.5; m/z (EI) 227.0218 (M^+^ - H^+^ for C_9_H_8_ClN_2_O_3_^-^ requires 227.0223).

**6c:** δ_H_ (500MHz; DMSO-d_6_) 11 (1 H, br s), 8.06 (1 H, d, *J* = 8.5), 7.72 (1 H, dd, *J =* 8.2, 8), 7.32 (1 H, d, J = 7.6), 3.60 (3 H, s), 2.69-2.67 (2 H, m), 2.61-2.58 (2 H, m); δ_C_ (125MHz; DMSO-d_6_) 172.5, 171, 152, 141.5, 138.5, 122.5, 112, 51.5, 30.5, 28; m/z (EI) 284.9875 (M^+^ - H^+^ for C_10_H_10_N_2_O_3_Br^-^ requires 284.9873); Melting points: 138^o^C.

**7c**: δ_H_ (500MHz; DMSO-d_6_) 12 (1 H, s), 10.5 (1 H, br s), 8.07 (1 H, d, *J =* 8.1), 7.72 (1 H, dd, *J* = 8.2, 7.9), 7.31 (1H, d, *J* = 7.9), 2.64-2.61 (4 H, m); δ_C_ (125MHz; DMSO-d_6_) 173.5, 171.5, 152.5, 141.5, 139, 122.5, 122, 31, 28.5; m/z (EI) 270.9713 (M^+^ - H^+^ for C_9_H_8_BrN_2_O_3_^-^ requires 270.9718).

**6d:** δ_H_ (500MHz; CDCl_3_) 8.16 (1 H, br s), 7.98 (1 H, d, *J* = 8.8), 7.59 (1 H, dd, *J =* 7.9, 7.8), 6.89 (1 H, d, *J* = 7.8), 3.72 (3 H, s), 2.77-2.70 (4 H, m), 2.45 (3 H, s); δ_C_ (125MHz; CDCl_3_) 173, 170, 156.5, 150.5, 138.5, 119, 110.5, 52, 32, 29, 23.5; m/z (EI) 245.0902 (M^+^ + Na^+^ for C_11_H_14_N_2_O_3_Na^+^ requires 245.0904).

**7d**: δ_H_ (500MHz; DMSO-d_6_) 11.0 (1 H, bs), 7.91-7.86 (2 H, m), 7.13-7.11 (1 H, m), 4.21 (1 H, bs), 2.72-2.66 (2H, m), 2.55-2.51 (2H, m), 2.49 (3H, s); δ_C_ (125MHz; DMSO-d_6_) 173.5, 171.5, 154, 150, 141, 119, 111.5, 31, 29, 22; m/z (EI) 207.0770 (M^+^ - H^+^ for C_13_H_13_NO_4_^-^ requires 207.0772).

**6e:** δ_H_ (500MHz; CDCl_3_) 8.32 (1 H, s), 8.13 (1 H, d, *J* = 9.5), 7.79 (1 H, d, *J =* 9.2), 3.73 (3 H, s), 2.78-2.71 (4H, m); δ_C_ (125MHz; CDCl_3_) 173, 170, 149.5 148.5, 140.5, 115, 114.5, 52, 31.5, 28.5; m/z (EI) 284.9875 (M^+^ - H^+^ for C_10_H_10_N_2_O_3_Br^-^ requires 284.9873).

**7e**: δ_H_ (500MHz; DMSO-d_6_) 12 (1 H, br s), 10.5 (1 H, br s), 8.43 (1 H, s), 8.06 (1 H, d, *J* = 9.5), 7.99 (1H, d, *J* = 9.2), 2.65-2.63 (2 H, m), 2.53-2.52 (2 H, m); δ_C_ (125MHz; DMSO-d_6_) 173.5, 171.5, 151, 148.5, 140.5, 115, 113, 31, 28.5; m/z (EI) 270.9713 (M^+^ - H^+^ for C_9_H_8_BrN_2_O_3_^-^ requires 270.9718).

**6f:** δ_H_ (500MHz; CDCl_3_) 7.95 (1 H, s), 3.72 (3 H, s), 2.76-2.70 (4 H, m), 2.57 (3 H, s), 2.40 (3H, s); δ_C_ (125MHz; CDCl_3_) 173, 170, 155.5, 150, 148.5, 118, 113, 52, 32, 29, 25, 23.5; m/z (EI) 337.0158 (M^+^ + Na^+^) for C_12_H_15_BrN_2_O_3_Na^+^ requires 337.02).

**7f**: δ_H_ (500MHz; DMSO-d_6_): 10.5 (1 H, s), 8.0 (1 H, s), 2.63-2.50 (4H, m), 2.35 (3H, s); δ_C_ (125MHz; DMSO-d_6_): 173.5, 171, 154.5, 150, 148.5, 116.5, 113, 31, 28.5, 24.5, 23; m/z (EI) 301.0182 (M^+^ + H^+^ for C_11_H_14_BrN_2_O_3_^+^ requires 299.0031).

**Legends to Scheme 3:** (a) **8** (1 eq), pyridine (1.2 eq), **2** (1.1 eq), MeCN, 0^o^C, 1 h, 80-85%; (b) **9** (1 eq), LiOH (1.2 eq), THF: H_2_O (4:1, 10 mL), RT, 2 h, 90-95%.

**Spectral Characterization of the compounds:**

**9a**: δ_H_ (500MHz; CDCl_3_) 7.85-7.81 (2H, m), 7.48-7.45 (1H, m), 7.36-7.33 (1H, m), 3.73 (3H, s), 2.86-2.79 (4H, m); δ_C_ (125MHz; CDCl_3_) 173, 170, 159, 147, 131.5, 126.5, 124, 121.5, 120.5, 52, 31, 28.5; m/z (EI) 263.0485 (M^+^ - H^+^ for C_12_H_11_N_2_O_3_S^-^ requires 263.0490).

**9b:** δ_H_ (500MHz; DMSO-d_6_) 12.4 (1 H, s), 7.96 (1 H, d, *J* = 8.8), 7.73 (1 H, d, *J =* 8.8), 7.43 (1 H, d, *J =* 8.5), 7.41 (1H, d, *J* = 8.2), 2.73 (2 H, dd, *J* 7, 7.3), 2.59 (2 H, dd, *J* 7.2, 7); δ_C_ (125MHz; DMSO-d_6_) 173.5, 171.5, 158, 148.5, 131.5, 126, 123.5, 121.5, 120.5, 30, 28; m/z (EI) 249.0328 (M^+^ - H^+^ for C_11_H_9_N_2_O_3_S^-^ requires 249.0334).

**10a:** δ_H_ (500MHz; CDCl_3_) 7.86-7.84 (1 H, m), 7.78-7.76 (1 H, m), 7.48-7.45 (1 H, m), 7.36-7.34 (1 H, m), 3.65 (3H, s), 2.61-2.58 (2 H, m), 2.43-2.40 (2 H, m), 2.10-2.04 (2H, m); δ_C_ (125MHz; CDCl_3_) 173, 171, 159, 147.5, 132, 126.5, 124, 121.5, 120.5, 51.5, 35, 32.5, 20; m/z (EI) 277.0641 (M^+^ - H^+^ for C_13_H_13_N_2_O_3_S^-^ requires 277.0647).

**10b:** δ_H_ (500MHz; DMSO-d_6_) 12.0 (1 H, br s), 7.97 (1 H, d, *J* = 8.9), 7.73 (1 H, d, *J =* 8.5), 7.43 (1 H, t, *J* = 8.5), 7.30 (1H, t, *J* = 8.5), 2.57-2.53 (2 H, m), 2.32-2.28 (2 H, m), 1.87-1.84 (2 H, m); δ_C_ (125MHz; DMSO-d_6_) 174, 171, 158, 148.5, 131.5 126, 123.5, 121.5, 120.5, 34, 33, 20; m/z (EI) 263.0485 (M^+^ - H^+^ for C_12_H_11_N_2_O_3_S^-^ requires 263.0490).

**Legends to Scheme 4:** (a) **11** (1 eq), pyridine (1.2 eq), **2** (1.1 eq), 0^o^C, 1 h, 70-75%; (b) **12** (1 eq), LiOH (1.2 eq), THF: H_2_O (4:1, 10 mL), RT, 2 h, 90-95%.

**Spectral Characterization of the compounds:**

**12a:** δ_H_ (500MHz; CDCl_3_) 10.0 (1 H, br s), 8.58 (1 H, d, *J* = 6.7), 8.15 (1 H, d, *J* 7.9), 7.87 (1 H, dd, *J* = 7.9, 6), 7.48 (1H, dd, *J* = 7.9, 6), 3.72 (3 H, s), 2.78-2.65 (4 H, m); δ_C_ (125MHz; CDCl_3_) 173, 168.5, 160.5, 148.5, 148, 137.5 127, 122.5, 52, 29, 28.5; m/z (EI) 274.0711 (M^+^ + H^+^ for C_11_H_13_N_3_O_4_Na^+^ requires 274.0828).

**13a:** δ_H_ (500MHz; DMSO-d_6_) 12 (1 H, br s), 10.5 (1 H, br s), 10 (1 H, br s), 8.68-8.66 (1 H, m), 8.03-8.02 (2H, m), 7.67-7.63 (1 H, m); δ_C_ (125MHz; DMSO-d_6_) 173.5, 170, 162.5, 149, 148.5, 137.5 127, 122.5, 28.5, 26; m/z (EI) 236.0666 (M^+^ - H^+^ for C_10_H_10_N_3_O_4_^-^ requires 236.0671).

**12b:** δ_H_ (500MHz; CDCl_3_) 10.3 (1 H, br s), 9.07 (1 H, br s), 8.58 (1 H, d, *J =* 7.9), 8.14 (1 H, d, *J* = 7.8), 7.89-7.86 (1H, m), 7.49-7.46 (1 H, m), 3.68 (3 H, s), 2.47-2.44 (4 H, m), 2.08-2.02 (2 H, m); δ_C_ (125MHz; CDCl_3_) 173.5, 169, 160, 148.5, 148, 137.5, 127, 122.5, 51.5, 33, 20.5; m/z (EI) 288.0955 (M^+^ + Na^+^) for C_12_H_15_N_3_O_4_Na^+^ requires 288.10).

**13b:** δ_H_ (500MHz; D_2_O + DMSO-d_6_) 8.58-8.57 (1 H, m), 7.99-7.95 (2 H, m), 7.57 (1 H, m), 2.32 (2 H, t, *J* = 6), 2.17 (2 H, t, *J* = 6), 1.84-1.83 (2 H, m); δ_C_ (125MHz; D_2_O + DMSO-d_6_) 182, 181, 176, 165.5, 149, 138.5, 127.5, 123, 28.5, 23.5, 22; m/z (EI) 250.0822 (M^+^ - H^+^ for C_11_H_12_N_3_O_4_^-^ requires 250.0833).

**Legends to Scheme 5:** (a) **14** (1 eq), Et_3_N (3 eq), PyBOP^®^ (1.5 eq), amine (1.2 eq), MeCN, RT, 5 h, 70-75% (b) TFA:DCM (1:1), 0^o^C to RT, 2 h, 90%, (c) **15c-d** (1 eq), pyridine (1.2 eq), **2** (1.1 eq), MeCN, 0^o^C, 1 h, 55-65%; (d) **16** (1 eq), LiOH (1.2 eq), THF: H_2_O (4:1, 10 mL), RT, 2 h, 90-95%.

**Spectral Characterization of the compounds:**

**15a:** δ_H_ (500MHz; CDCl_3_) 9.68 (1 H, br s), 9.17 (1 H, s), 8.60 (1 H, s), 8.05 (1 H, d, J = 6.4), 7.99 (2 H, d, J = 6.4), 7.80-7.73 (2 H, m), 1.52 (9 H, s); δ_C_ (125MHz; CDCl_3_) 163.5, 155, 151.5, 142, 135.5, 131, 130, 129.5, 11928, 127.5, 121, 81.5, 28; m/z (EI) 310.1162 (M^+^ + Na^+^ for C_15_H_17_N_3_NaO_3_^+^ requires 310.1168).

**15b:** δ_H_ (500MHz; CDCl_3_) 8.56 (1 H, br s), 8.31 (1 H, s), 7.84-7.80 (4 H, m), 7.57-7.50 (2 H, m), 6.90 (1 H, m), 1.51 (9 H, s); δ_C_ (125MHz; CDCl_3_) 167, 156, 135, 132.5, 129, 128.5, 128, 127.5, 126.5, 123.5, 82, 28.5; m/z (EI) 309.1210 (M^+^ + Na^+^ for C_16_H_18_N_2_NaO_3_^+^ requires 309.1215).

**16b:** δ_H_ (500MHz; DMSO-d_6_) 10.5 (1 H, s), 10.04 (1 H, s), 8.50 (1 H, br s), 8.05-7.94 (4 H, m), 7.65-7.61 (2 H, m), 3.61 (3 H, s), 2.59-2.50 (4 H, m); δ_C_ (125MHz; DMSO-d_6_) 172.5, 170, 165.5, 134.5, 132, 129.5, 129, 128.5, 128, 127.5, 127, 126.5, 124, 51, 28.5, 27.5; m/z (EI) 323.1002 (M^+^ + Na^+^ for C_16_H_16_N_2_NaO_4_^+^ requires 323.1008).

**17b:** δ_H_ (500MHz; DMSO-d_6_) 10.5 (1 H, br s), 10.02 (1 H, br s), 8.50 (1 H, br s), 8.50 (1 H, br s), 8.10-7.93 (3 H, m), 7.66-7.61 (2 H, m), 2.52-2.46 (4 H, m); δ_C_ (125MHz; DMSO-d_6_) 173.5, 170.5, 165.5, 134.5, 132, 130, 129, 129, 128.5, 128, 127, 124, 123.5, 29, 28.5; m/z (EI) 309.0851 (M^+^ + Na^+^ for C_15_H_14_N_2_NaO_4_^+^ requires 309.0851).

**Legends to Scheme 6:** (a) **18** (1 eq), Et_3_N (3 eq), PyBOP^®^ (1.5 eq), amine-ester hydrochloride (1.2 eq), MeCN, RT, 5 h, 70-75% (b) **19** (1 eq), LiOH (1.2 eq), THF: H_2_O (4:1, 10 mL), RT, 2 h, 90-95%.

**Spectral Characterization of the compounds:**

**19:** δ_H_ (500MHz; DMSO-d_6_) 10 (1 H, s), 9.0 (1 H, brs), 8.5 (1 H, s), 8.26 (1 H, d, *J* = 9.1), 8.2 (1H, d, *J* = 8.2), 7.9-7.87 (1 H, m), 7.84-7.80 (2 H, m), 3.64 (1 H, m), 3.63 (3 H, s), 3.61-3.60 (1 H, m), 2.68-2.66 (2 H, m); δ_C_ (125MHz; DMSO-d_6_) 172, 164, 151.5, 143.5, 135.5, 131.5, 129.5, 129, 128, 127.5, 119.5, 51.5, 35, 33.5; m/z (EI) 281.0900 (M^+^ + H^+^ for C_14_H_14_N_2_O_3_Na^+^ requires 281.0902).

**20:** δ_H_ (500MHz; DMSO-d_6_) 12.5 (1 H, brs), 9.5 (1 H, s), 8.95 (1 H, t, *J =* 6.3), 8.57 (1 H, s), 8.27-8.20 (2H, m), 7.9-7.78 (2 H, m), 3.66-3.57 (2 H, m), 2.65-2.60 (2 H, m); δ_C_ (125MHz; DMSO-d_6_) 173, 164, 151.5, 143.5, 135.5, 131.5, 129.5, 129, 128, 127.5, 119.5, 35, 34; m/z (EI) 243.0764 (M^+^ - H^+^ for C_13_H_11_N_2_O_3_^-^ requires 243.077).

**Legends to Scheme 7:** (a) **21** (1 eq), pyridine (1.2 eq), **2** (1.1 eq), 0^o^C, 1 h, 55-65%; (b) **22** (1 eq), LiOH (1.2 eq), THF: H_2_O (4:1, 10 mL), RT, 2 h, 90-95%.

**Spectral Characterization of the compounds:**

**22:** δ_H_ (500MHz; CDCl_3_) 8.34 (1 H, d, J = 10.1), 8.22 (1 H, d, *J* = 9.5), 7.83 (1 H, d, *J =* 8.8), 7.81 (1 H, d, *J* = 8.5), 7.70 (1H, dd, *J* = 8.5, 7.3), 7.47 (1 H, dd, *J* = 8.2, 7.2), 3.73 (3 H, s), 2.82-2.77 (4 H, m); δ_C_ (125MHz; CDCl_3_) 173, 171, 150.5, 145.5, 139.5, 130.5, 127.5, 126.5, 126, 125.5, 114, 52, 32, 28.5; m/z (EI) 259.1083 (M^+^ + H^+^ for C_14_H_15_N_2_O_3_^+^ requires 259.1078).

**23**_:_ δ_H_ (500MHz; DMSO-d_6_) 10.5 (1 H, brs), 8.34 (1 H, d, *J* = 9.5), 8.27 (1 H, d, *J* 9.5), 7.91 (1 H, d, *J* = 8.5), 7.80 (1H, d, *J* = 8.9), 7.71 (1 H, dd, *J* = 8.5), 7.49 (1 H, dd, *J =* 8.2), 2.72 (2 H, m), 2.56-2.54 (2 H, m); δ_C_ (125MHz; DMSO-d_6_) 174, 171.5, 151.5, 146.5, 138, 130, 127.5, 126.5, 125.5, 124.5, 114, 31, 28.5; m/z (EI) 243.0770 (M^+^ - H^+^) for C_13_H_11_N_2_O_3_^+^ requires 243.0774); Melting points: 191^o^C.

**Legends to Scheme 8:** (a) **24** (1 eq), Et_3_N (3 eq), PyBOP^®^ (1.5 eq), amine-ester hydrochloride (1.2 eq), MeCN, RT, 5 h, 70-75% (b) **25** or **27** (1 eq), LiOH (1.2 eq), THF: H_2_O (4:1, 10 mL), RT, 2 h, 90-95%.

**Spectral Characterization of the compounds:**

**25:** δ_H_ (400MHz; CDCl_3_) 8.30 (1 H, t, *J* = 5.2), 8.14-8.09 (2 H, m), 7.53-7.49 (1 H, m), 7.32-7.26 (1 H, m), 7.20 (1 H, d, *J* = 7.6), 4.33 (2 H, d, *J* = 4.4), 3.80 (3 H, s); δ_C_ (100MHz; CDCl_3_) 171, 164.5, 152.5, 147, 137.5, 136.5, 130, 129.5, 119.5, 118, 111.5, 52.5, 41.5; m/z (EI) 261.0876 (M^+^ + H^+^ for C_13_H_13_N_2_O_4_^+^ requires 261.0875).

**26:** δ_H_ (400MHz; DMSO-d_6_) 12.75 (1 H, brs), 10.14 (1 H, d, *J* = 6.5), 9.97 (1 H, d, *J* = 6.4), 8.51 (1 H, dd, *J* = 8.4, 6.8), 8.13 (1 H, dd, *J* = 8, 6.8), 7.60-7.20 (2 H, m), 7.18-7.17 (1 H, m), 4.10 (2 H, d, *J* = 6); δ_C_ (100MHz; DMSO-d_6_) 172, 165, 154.5, 148, 139, 130.5, 121, 120, 119.5, 118.5, 112.5, 41; m/z (EI) 245.0558 (M^+^ - H^+^ for C_12_H_9_N_2_O_4_^-^ requires 245.0562).

**27:** δ_H_ (400MHz; CDCl_3_) 8.73 (1 H, m), 8.33-8.32 (2 H, m), 7.58-7.53 (1 H, m), 7.41-7.39 (1 H, m), 7.28-7.24 (1 H, m), 3.83-3.82 (2 H, m), 3.74 (3 H, s), 2.74 (brs, 2H); δ_C_ (100MHz; CDCl_3_) 173, 164, 152.5, 147.5, 138, 136.5, 130, 129.5, 119.5, 118, 111.5, 52, 35, 34; m/z (EI) 297.0846 (M^+^ + Na^+^ for C_14_H_14_N_2_NaO_4_^+^ requires 297.09).

**28:** δ_H_ (400MHz; DMSO-d_6_) 12.30 (1 H, brs), 10.15 (1 H, brs), 9.69 (1 H, brs), 8.51-8.48 (1 H, m), 8.14-8.11 (1 H, m), 7.58-7.53 (1 H, m), 7.49-7.46 (1 H, m), 7.18-7.16 (1 H, m), 3.62 (2 H, m), 2.63-2.60 (2 H, m); δ_C_ (100MHz; DMSO-d_6_) 171, 164.5, 152.5, 147, 137.5, 136.5, 130, 129.5, 119.5, 118, 111.5, 52.5, 41.5; m/z (EI) 283.0689 (M^+^ + H^+^ for C_13_H_12_N_2_NaO_4_^+^ requires 283.07).

**30a** (Methyl)**:** (400MHz; CDCl_3_) 8.99 (s, 1H), 8.65 (s, 1H), 8.41 (br s, 1H) 7.94 (d, 1H, J = 8 Hz), 7.88 (d, 1H, J = 8 Hz), 7.33, (t, 1H, J = 8 Hz), 3.91 (s, 3H); δ_C_ (100MHz; CDCl_3_) 153.6, 148.1, 137.3, 134.5, 127.28, 126.9, 125.7, 121.4, 105.1, 52.6; m/z (EI) 302.9741 (M^+^ + Na^+^ for C_11_H_9_BrN_2_NaO_2_ requires 302.9740).

**30b** (Methyl)**:** δ_H_ (400MHz; CDCl_3_) 9.23 (s, 1H) 8.74 (s, 1H), 8.17 (d, 1H, J = 8.5 Hz), 8.03 (d, 1H, J = 8.5 Hz), 7.89 (t, 1H, J = 7.5 Hz), 7.68 (t, 1H, J = 7.5 Hz), 3.73 (s, 3H), 2.97 (t, 2H, J = 6.5 Hz) 2.85 (t, 2H, J = 6.5 Hz); δ_C_ (100MHz; CDCl_3_) 173.3, 170.3, 155.5, 148.9, 136.6, 133.5, 129.5, 128.4, 127.9, 125.5, 124.1, 114.5, 52.1, 31.9, 29.0; m/z (EI) 284.16 [M+H]+ for C_15_H_14_N_3_O_3_ requires 284.10).

**Supplementary Figures**

Figure S1. Inhibition of 2OG-oxygenases by succinamic acid derivatives (SCAs). (**A**) Structures of selected tested SCAs. SCAs for which crystal structures were obtained with nPHD2 are boxed in red. (**B)** The SCAs, with and without pre-incubation, showed only moderate to weak inhibition of nPHD2 using a time-resolved fluorescence resonance energy transfer assay[^20^](#_ENREF_20) with a 60 min pre-incubation. Consistent with the *in vitro* assay results, representative compounds (methyl esters of **4a**, **4c**, **9b** and **7f**) displayed very low levels of activity in human Hep3B cell lines (data not shown). Selected compounds were also tested against structurally related histone lysine demethylases (KDM2/ FBXL11, KDM4A and KDM7/ PHF8) using an MS-based assay[^21^](#_ENREF_21). The most potent nPHD2 inhibitor, **4a** only weakly inhibited PHF8 (IC_50_, ~295 μM) and FBXL11, and had (almost) no effect on KDM4A even at a 1:1000 enzyme: compound ratio. Figures **C**-**F** compare active site close-up views from the nPHD2.FG2216 and nPHD2.SCA crystal structures. nPHD2 crystallizes with selected SCAs in the reported *P*6_3_ form (see Fig.1). Like FG2216 (**c**), SCAs coordinate the PHD2 active site metal ion (Fe^II^) via the heteroaromatic ring nitrogen and the amide carbonyl oxygen. Whereas the bidentate coordination of Fe(II) by PHD inhibitors, FG2216/ UN9 (PDB: 4BQX)[^14^](#_ENREF_14), FG2216Ala/ FNT (PDB: 4BQY)[^14^](#_ENREF_14), quinoline-N-Me/ QNM (PDB: 4BQW)[^14^](#_ENREF_14) and JNJ41536014 (PDB: 3OUH)[^22^](#_ENREF_22) form an approximately planar five-membered chelate ring (**c**), the SCAs form a six-membered ring (**D**-**F**). The chelate ring size may affect the stability of the enzyme-inhibitor complex as proposed with other dioxygenases[^23^](#_ENREF_23). The carboxylate sidechain (2OG C5-equivalent) of the SCAs (i.e. C14 of FG2216 and C15 of **4a**) is positioned to form electrostatic interactions with the Arg383 guanidino group and hydrogen bond with the phenolic OH of Tyr329. By contrast with FG2216, the C13 and C14 atoms of the SCAs undergo apparent twisting to accommodate their additional side chain carbon. Note, the SCAs have four bonds capable of facile rotation, compared to three such bonds for FG2216 (and related PHD inhibitors).

| **A** | 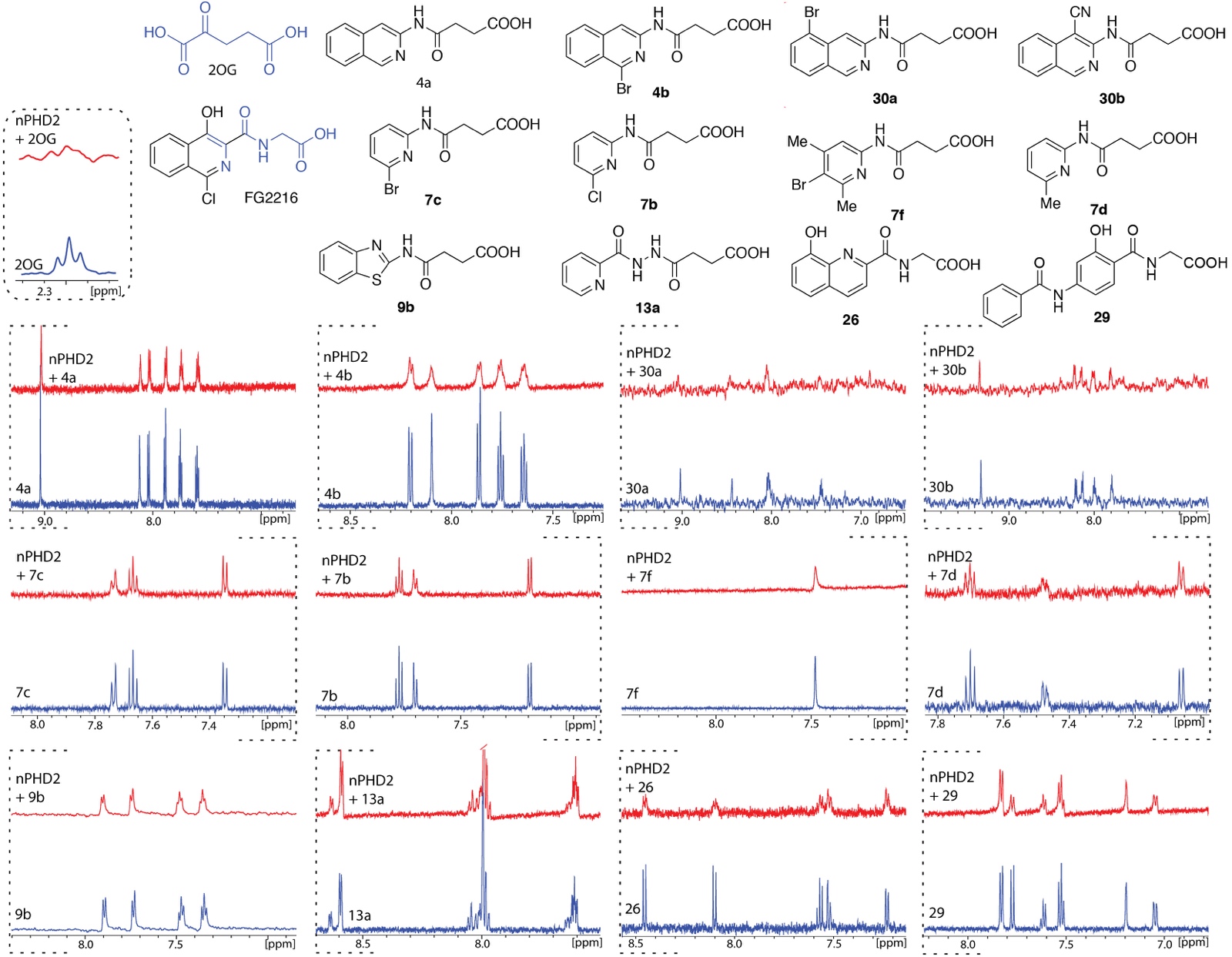 |
| --- | --- |
| **B** | 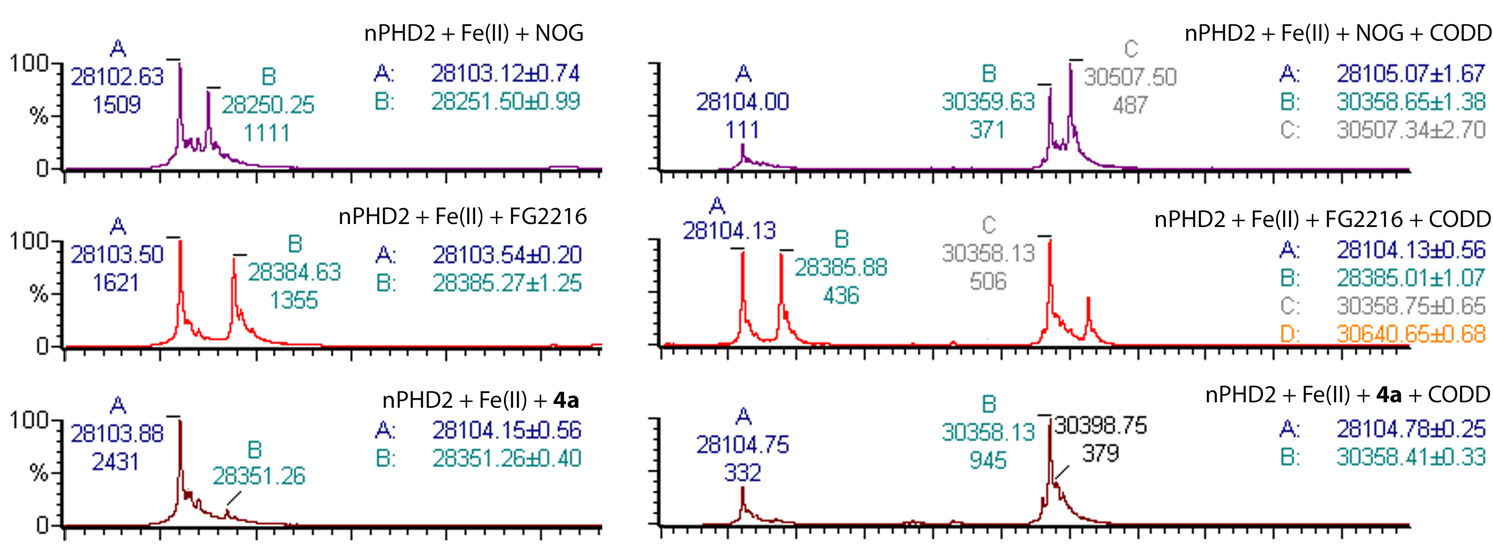 |

Figure S2. NMR and MS analysis of PHD2.SCA complexes. (**A**) Binding by NMR is detected by a signal intensity reduction and line broadening in the CPMG analyses and a change in intensity/ sign of the NOE response in the wLOGSY analyses[^8^](#_ENREF_8)^,^[^9^](#_ENREF_9). In most cases, the addition of nPHD2 to the SCAs leads to little line broadening and partial signal reduction of the compound peaks, indicating that, within the limits of detection, the compounds studied bind only weakly to nPHD2 in solution. Those observations are consistent with wLOGSY analyses which also indicate a weak binding event. Note wLOGSY spectra could not be recorded for all compounds because of solubility issues and the formation of aggregates in solution (data not shown). Assay mixtures were buffered in 50 mM Tris-D_11_, pH 7.5, in 10 % D_2_O and 90 % H_2_O containing 50 μM Zn(II). (**B**) MS analyses suggest that, compared to NOG and FG2216, which are relatively potent PHD inhibitors, the SCA **4a** forms only a weak complex with nPHD2, either alone (left) or in the presence of HIF-1α CODD (right) (mass units: Dalton).

###

### **Figure S3. PHD2 lysine-residue methylation**. *N*^ε^-Lysine methylation of nPHD2 was carried out in an attempt to obtain different crystal packing from that of the *P*6_3_ form. nPHD2-Me retains substantial activity compared to nPHD2 when assayed monitoring turnover of [^14^C]-labelled 2OG (A) or monitoring conversion of 2OG to succinate by ^1^H NMR[^8^](#_ENREF_8)^,^[^9^](#_ENREF_9) (B). The latter assays suggest NODD turnover may be more affected by methylation than CODD turnover. MS based assays with NODD or CODD peptides (for 20 min) alone (C) or in competition (D) reveal nPHD2-Me catalyses hydroxylation of NODD and CODD to similar extent as wildtype nPHD2. (E) Non-denaturing MS analyses suggest that, nPHD2-Me forms a weaker complex with NODD compared to CODD. (F) Despite apparent modification of all 21 lysines, nPHD2-Me still crystallizes in the *P*6_3_ form when complexed with 4a, as observed for nPHD2 (G); methylation improved the electron density maps for some of the lysine side chains. (H) Analysis of the nPHD2.NODD complex crystal structure[^2^](#_ENREF_2) reveals that PHD2 K244 and K297 (boxed hashed black) make hydrophobic interactions with the NODD LXXLAP motif and *C*-terminal region Ile408, respectively. It is possible that *N*ε-methylation of these two lysine residues (and possibly others) alters the hydrophobic contacts with NODD by nPHD2-Me relative to unmodified nPHD2. See Supplementary Methods for assay details.


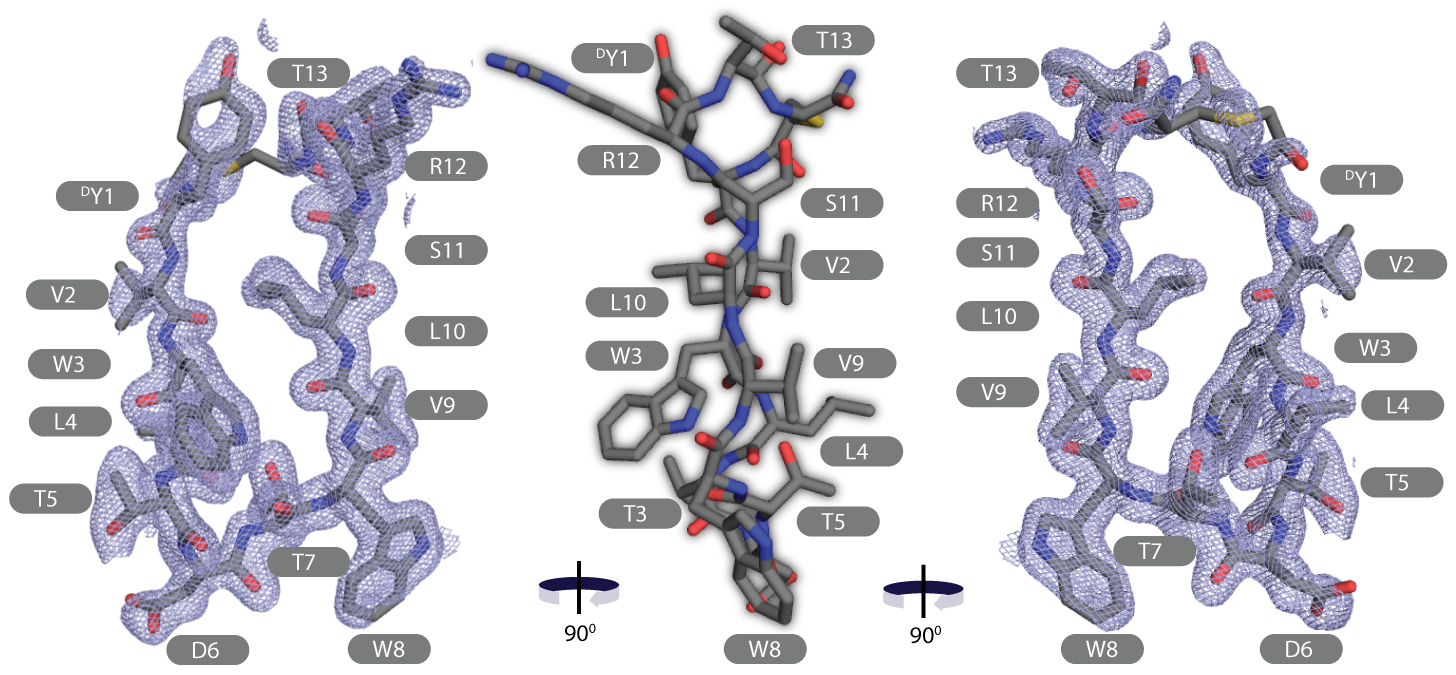


### **Figure S4. The 3C peptide conformation as observed in the cPHD2.3C complexes.** 3C adopts a planar overall conformation approximating to a rectangle with ^D^-Y1-D6 forming one long side and W8-R12 the other. The 3C ring is relatively flat when viewed side-on (middle panel) with the two long sides of the rectangle eclipsing each other. On the short edges this planar arrangement is disrupted and the side chains extend away from the plane.

Figure S5. Polar interactions between the cyclic peptide 3C and cPHD2 N/C-terminal residues. The interacting residues of two PHD2 monomers in the crystalline lattice are labelled with their numbers with colouring according to which monomer they are from (green or purple**)**. Water molecules are shown as red spheres; hydrogen bonds are shown as dashed black lines. 3C forms a β-sheet with residues in both the nPHD2 monomers; the two strands within the peptide do not interact with each other directly via their backbones. There are additional backbone to side-chain interactions between ^PHD2^L188 and ^PHD2^F213 with ^3C^R6 and ^PHD2^A399 with ^3C^T5, as well as side-chain to side-chain interactions between ^PHD2^D212 and ^3C^T13 / ^3C^S11, and ^PHD2^K186 with 3C ^D^-Y1.

**Table S1. Data collection and refinement statistics of the nPHD2.SCA complexes.**

|  | **nPHD2.4a** | **nPHD2.7f** | **nPHD2.30a** | **nPHD2-Me.4a** |
| --- | --- | --- | --- | --- |
|  |  |  |  |  |
| **PDB acquisition codes** | 6YVX | 6YVW | 6YVZ | 6YW0 |
|  |  |  |  |  |
| **Data collection** |  |  |  |  |
| Beamline (Wavelength, Å) | DLS I02 (0.9800) | In-house (1.5418) | DLS I02 (0.9800) | ESRF ID232 (0.8726) |
| Detector | Pilatus 6M-F | CCD SATURN 944 | Pilatus 6M-F | MARMOSAIC 225 |
| Data processing | XDS[^24^](#_ENREF_24), SCALA[^25^](#_ENREF_25) | HKL2000[^26^](#_ENREF_26) | XDS[^24^](#_ENREF_24), SCALA[^25^](#_ENREF_25) | HKL2000[^26^](#_ENREF_26) |
| Space group | *P*6_3_ | *P*6_3_ | *P*6_3_ | *P*6_3_ |
| Cell dimensions |  |  |  |  |
| *a*, *b*, *c* (Å) | 109.95 109.95, 39.22 | 110.17, 110.17, 39.79 | 109.88, 109.88, 39.08 | 110.93, 110.93, 39.66 |
| *α*, *β*, *γ* (°) | 90, 90, 120 | 90, 90, 120 | 90, 90, 120 | 90, 90, 120 |
| No. of molecules/ ASU | 1 | 1 | 1 | 1 |
| No. reflections | 25415 (3660)* | 19800 (968)* | 21231 (1587)* | 14427 (2080)* |
| Resolution (Å) | 54.97-1.80  (1.90-1.80)* | 18.45-1.97  (2.00-1.97)* | 36.15-1.91  (1.96-1.91)* | 36.31-2.20  (2.32-2.20)* |
| *R*_sym_ or *R*_merge_** | 0.088 (1.319)* | 0.092 (0.915)* | 0.091 (1.273)* | 0.177 (2.037)* |
| *I*/σ*I* | 13.1 (2.4)* | 18.8 (2.5)* | 14.5 (2.1)* | 19.1 (2.1)* |
| Completeness (%) | 100 (100)* | 99.8 (99.6)* | 99.9 (100)* | 99.9 (100)* |
| Redundancy | 11.3 (11.4)* | 7.8 (7.2)* | 11.4 (12.0)* | 20.3 (20.8)* |
| CC (1/2) | 0.998 (0.718)* | 0.990 (0.820)* | 0.998 (0.662)* | 0.990 (0.690)* |
| Wilson *B* value (Å^2^) | 31.2 | 36.0 | 37.2 | 41.0 |
|  |  |  |  |  |
| **Refinement** |  |  |  |  |
| *R*_work/_ *R*_free_^‡^ | 0.160/0.189 | 0.188/0.208 | 0.168/0.204 | 0.192/0.221 |
| No. atoms |  |  |  |  |
| -Enzyme | 1776 | 1777 | 1739 | 1757 |
| -Metal | 1 | 1 | 1 | 1 |
| -Ligand | 18 (**4a**) | 17 (**7f**) | 19 (**30a**) | 18 (**4a**) |
| -Water | 128 | 122 | 89 | 63 |
| B-factors |  |  |  |  |
| -Enzyme | 44.4 | 53.4 | 52.1 | 65.4 |
| -Metal | 24.8 | 28.5 | 27.9 | 32.9 |
| -Ligand | 40.5 | 69.9 | 70.4 | 38.9 |
| -Water | 53.1 | 54.4 | 58.8 | 57.8 |
| R.m.s deviations |  |  |  |  |
| -Bond lengths (Å) | 0.014 | 0.017 | 0.014 | 0.004 |
| -Bond angles (º) | 1.202 | 1.439 | 1.173 | 0.851 |
|  |  |  |  |  |

*Highest resolution shell shown in parentheses.

**R_sym_ = ∑|*I*-<*I*>|/∑*I*, where *I* is the intensity of an individual measurement and <*I*> is the average intensity from multiple observations.

^‡^R_factor_ = ∑*_hkl_*||*F*_obs_(*hkl*)| − k |*F_c_*_alc_(*hkl*)||/ ∑*_hkl_*|*F*_obs_(*hkl*)| for the working set of reflections; R_free_ is the R_factor_ for ~5% of the reflections excluded from refinement.

**Table S2. Data collection and refinement statistics of the cPHD2.3C complexes.**

|  | **cPHD2.2OG.3C** | **cPHD2.NOG.3C** | **cPHD2.FG2216.3C** | **cPHD2.NOG.CODD.3C** |
| --- | --- | --- | --- | --- |
|  |  |  |  |  |
| **PDB acquisition codes** | 6YW1 | 6YW4 | 6YW2 | 6YW3 |
|  |  |  |  |  |
| **Data collection** |  |  |  |  |
| Beamline (Wavelength, Å) | DLS I04 (0.9795) | DLS I02 (0.9795) | DLS I03 (0.9762) | DLS I03 (0.9762) |
| Detector | Pilatus 6M-F | Pilatus 6M-F | Pilatus 6M-F | Pilatus 6M-F |
| Data processing | HKL2000[^26^](#_ENREF_26) | HKL2000[^26^](#_ENREF_26) | XDS[^24^](#_ENREF_24), SCALA[^25^](#_ENREF_25) | XDS[^24^](#_ENREF_24), SCALA[^25^](#_ENREF_25) |
| Space group | *P*6_5_ | *P*6_5_ | *P*6_5_ | *P*2_1_2_1_2 |
| Cell dimensions |  |  |  |  |
| *a*, *b*, *c* (Å) | 46.53, 46.53, 202.35 | 46.62, 46.62, 203.71 | 46.18, 46.18, 202.26 | 76.44, 81.29, 43.22 |
| *α*, *β*, *γ* (°) | 90, 90, 120 | 90, 90, 120 | 90, 90, 120 | 90, 90, 90 |
| No. of molecules/ ASU | 1 | 1 | 1 | 1 |
| No. reflections | 42887 (4250)* | 37807 (3124)* | 13502 (1362)* | 12861 (1830)* |
| Resolution (Å) | 50.0-1.46  (1.51-1.46)* | 50.0-1.53  (1.58-1.53)* | 101.13-2.14  (2.26-2.14)* | 76.39-2.28  (2.40-2.28)* |
| *R*_sym_ or *R*_merge_** | 0.060 (1.108)* | 0.127 (1.750)* | 0.168 (1.341)* | 0.164 (1.043)* |
| *I*/σ*I* | 28.4 (1.5)* | 20.1 (1.7)* | 12.6 (2.2)* | 9.6 (2.3)* |
| Completeness (%) | 100 (100)* | 100 (100)* | 100 (100)* | 100 (100)* |
| Redundancy | 7.6 (7.2)* | 16.0 (13.1)* | 15.4 (15.8)* | 9.1 (9.0)* |
| CC (1/2) | 0.990 (0.617)* | 0.990 (0.653)* | 0.998 (0.723)* | 0.996 (0.700)* |
| Wilson *B* value (Å^2^) |  |  |  |  |
|  |  |  |  |  |
| **Refinement** |  |  |  |  |
| *R*_work/_ *R*_free_^‡^ | 0.164/0.173 | 0.164/0.177 | 0.175/0.192 | 0.187/0.219 |
| No. atoms^ψ^ |  |  |  |  |
| -Enzyme | 1662 | 1659 | 1615 | 1715 |
| -Metal | 1 | 1 | 1 | 1 |
| -Ligand | 10 (2OG) | 10 (NOG) | 19 (FG2216) | 10 (NOG) |
| -Substrate | - | - | - | 137 |
| -Cyclic peptide | 126 | 126 | 126 | 126 |
| -Water | 222 | 190 | 90 | 103 |
| B-factors |  |  |  |  |
| -Enzyme | 30.9 | 31.2 | 43.8 | 35.1 |
| -Metal | 18.1 | 16.9 | 24.0 | 16.1 |
| -Ligand | 21.6 | 19.8 | 36.2 | 25.3 |
| -Substrate | - | - | - | 47.3 |
| -Cyclic peptide | 36.2 | 33.1 | 47.4 | 35.1 |
| -Water | 40.2 | 32.7 | 45.3 | 35.8 |
| R.m.s deviations |  |  |  |  |
| -Bond lengths (Å) | 0.006 | 0.017 | 0.004 | 0.005 |
| -Bond angles (º) | 0.917 | 1.356 | 0.933 | 0.804 |
|  |  |  |  |  |

*Highest resolution shell shown in parentheses.

**R_sym_ = ∑|*I*-<*I*>|/∑*I*, where *I* is the intensity of an individual measurement and <*I*> is the average intensity from multiple observations.

^‡^R_factor_ = ∑*_hkl_*||*F*_obs_(*hkl*)| − k |*F_c_*_alc_(*hkl*)||/ ∑*_hkl_*|*F*_obs_(*hkl*)| for the working set of reflections; R_free_ is the R_factor_ for ~5% of the reflections excluded from refinement.

**Supplementary References**

1 Chowdhury, R. *et al.* Structural basis for binding of hypoxia-inducible factor to the oxygen-sensing prolyl hydroxylases. *Structure* **17**, 981-989 (2009).

2 Chowdhury, R. *et al.* Structural basis for oxygen degradation domain selectivity of the HIF prolyl hydroxylases. *Nat Commun* **7**, 12673, doi:10.1038/ncomms12673 (2016).

3 Walter, T. S. *et al.* Lysine methylation as a routine rescue strategy for protein crystallization. *Structure* **14**, 1617-1622 (2006).

4 McAllister, T. E. *et al.* Non-competitive cyclic peptides for targeting enzyme–substrate complexes. *Chem. Sci.*, doi:10.1039/C8SC00286J (2018).

5 Dao, J. H. *et al.* Kinetic characterization and identification of a novel inhibitor of hypoxia-inducible factor prolyl hydroxylase 2 using a time-resolved fluorescence resonance energy transfer-based assay technology. *Anal Biochem* **384**, 213-223 (2009).

6 Chan, M. C. *et al.* Potent and Selective Triazole-Based Inhibitors of the Hypoxia-Inducible Factor Prolyl-Hydroxylases with Activity in the Murine Brain. *PloS one* **10**, e0132004, doi:10.1371/journal.pone.0132004 (2015).

7 Mecinovic, J. *et al.* ESI-MS studies on prolyl hydroxylase domain 2 reveal a new metal binding site. *ChemMedChem* **3**, 569-572 (2008).

8 Abboud, M. I. *et al.* Studies on the Substrate Selectivity of the Hypoxia-Inducible Factor Prolyl Hydroxylase 2 Catalytic Domain. *Chembiochem* **19**, 2262-2267, doi:10.1002/cbic.201800246 (2018).

9 Abboud, M. I. *et al.* 2-Oxoglutarate regulates binding of hydroxylated hypoxia-inducible factor to prolyl hydroxylase domain 2. *Chem Commun (Camb)* **54**, 3130-3133, doi:10.1039/c8cc00387d (2018).

10 Dalvit, C., Fogliatto, G., Stewart, A., Veronesi, M. & Stockman, B. WaterLOGSY as a method for primary NMR screening: practical aspects and range of applicability. *J Biomol NMR* **21**, 349-359, doi:10.1023/a:1013302231549 (2001).

11 Huang, R., Bonnichon, A., Claridge, T. D. & Leung, I. K. Protein-ligand binding affinity determination by the waterLOGSY method: An optimised approach considering ligand rebinding. *Sci Rep* **7**, 43727, doi:10.1038/srep43727 (2017).

12 Roach, P. L. *et al.* Anaerobic crystallisation of an isopenicillin N synthase.Fe(II).substrate complex demonstrated by X-ray studies. *Eur J Biochem* **242**, 736-740, doi:10.1111/j.1432-1033.1996.0736r.x (1996).

13 McCoy, A. J. *et al.* Phaser crystallographic software. *J. Appl. Crystallogr.* **40**, 658-674, doi:10.1107/S0021889807021206 (2007).

14 Chowdhury, R. *et al.* Selective small molecule probes for the hypoxia inducible factor (HIF) prolyl hydroxylases. *ACS Chem Biol* **8**, 1488-1496, doi:10.1021/cb400088q (2013).

15 Adams, P. D. *et al.* PHENIX: a comprehensive Python-based system for macromolecular structure solution. *Acta Crystallogr. D Biol. Crystallogr.* **66**, 213-221, doi:10.1107/S0907444909052925 (2010).

16 Brunger, A. T. *et al.* Crystallography & NMR system: A new software suite for macromolecular structure determination. *Acta Crystallogr. D Biol. Crystallogr.* **54**, 905-921 (1998).

17 Smart, O. S. *et al.* Exploiting structure similarity in refinement: automated NCS and target-structure restraints in BUSTER. *Acta Crystallogr. D Biol. Crystallogr.* **68**, 368-380, doi:10.1107/S0907444911056058 (2012).

18 Emsley, P. & Cowtan, K. Coot: model-building tools for molecular graphics. *Acta Crystallogr. D Biol. Crystallogr.* **60**, 2126-2132 (2004).

19 Chen, V. B. *et al.* MolProbity: all-atom structure validation for macromolecular crystallography. *Acta Crystallogr. D Biol. Crystallogr.* **66**, 12-21, doi:10.1107/S0907444909042073 (2010).

20 Tegley, C. M. *et al.* Discovery of novel hydroxy-thiazoles as HIF-alpha prolyl hydroxylase inhibitors: SAR, synthesis, and modeling evaluation. *Bioorg Med Chem Lett* **18**, 3925-3928 (2008).

21 Kawamura, A. *et al.* Highly selective inhibition of histone demethylases by de novo macrocyclic peptides. *Nat Commun* **8**, 14773, doi:10.1038/ncomms14773 (2017).

22 Rosen, M. D. *et al.* Benzimidazole-2-pyrazole HIF Prolyl 4-Hydroxylase Inhibitors as Oral Erythropoietin Secretagogues. *ACS Med Chem Lett* **1**, 526-529, doi:10.1021/ml100198y (2010).

23 Merkel, M. *et al.* Chelate ring size variations and their effects on coordination chemistry and catechol dioxygenase reactivity of iron(III) complexes. *Inorg Chem* **44**, 7582-7589 (2005).

24 Kabsch, W. Integration, scaling, space-group assignment and post-refinement. *Acta Crystallogr. D Biol. Crystallogr.* **66**, 133-144 (2010).

25 Winn, M. D. *et al.* Overview of the CCP4 suite and current developments. *Acta Crystallogr. D Biol. Crystallogr.* **67**, 235-242, doi:10.1107/S0907444910045749 (2011).

26 Otwinowski, Z. & Minor, W. Processing of X-ray diffraction data collected in oscillation mode. *Method Enzymol.* **276**, 307-326, doi:Doi 10.1016/S0076-6879(97)76066-X (1997).
